# Advances in Designing and Developing Vaccines, Drugs and Therapeutic Approaches to Counter Human Papilloma Virus

**DOI:** 10.3389/fimmu.2018.02478

**Published:** 2018-11-12

**Authors:** Maryam Dadar, Sandip Chakraborty, Kuldeep Dhama, Minakshi Prasad, Rekha Khandia, Sameer Hassan, Ashok Munjal, Ruchi Tiwari, Kumaragurubaran Karthik, Deepak Kumar, Hafiz M. N. Iqbal, Wanpen Chaicumpa

**Affiliations:** ^1^Razi Vaccine and Serum Research Institute, Agricultural Research, Education and Extension Organization, Karaj, Iran; ^2^Department of Veterinary Microbiology, College of Veterinary Sciences and Animal Husbandry, West Tripura, India; ^3^Division of Pathology, ICAR-Indian Veterinary Research Institute, Bareilly, India; ^4^Department of Animal Biotechnology, LLR University of Veterinary and Animal Sciences, Hisar, India; ^5^Department of Genetics, Barkatullah University, Bhopal, India; ^6^Department of Biomedical Informatics, National Institute for Research in Tuberculosis, Indian Council of Medical Research, Chennai, India; ^7^Department of Veterinary Microbiology and Immunology, College of Veterinary Sciences, U P Pt. Deen Dayal Upadhayay Pashu Chikitsa Vigyan Vishwavidyalay Evum Go-Anusandhan Sansthan, Mathura, India; ^8^Central University Laboratory, Tamil Nadu Veterinary and Animal Sciences University, Chennai, India; ^9^Division of Veterinary Biotechnology, ICAR-Indian Veterinary Research Institute, Bareilly, India; ^10^Tecnologico de Monterrey, School of Engineering and Sciences, Monterrey, Mexico; ^11^Department of Parasitology, Center of Research Excellence on Therapeutic Proteins and Antibody Engineering, Faculty of Medicine SIriraj Hospital, Mahidol University, Bangkok, Thailand

**Keywords:** human papilloma virus (HPV), prophylaxis, vaccines, drugs, therapy

## Abstract

Human papillomavirus (HPV) is a viral infection with skin-to-skin based transmission mode. HPV annually caused over 500,000 cancer cases including cervical, anogenital and oropharyngeal cancer among others. HPV vaccination has become a public-health concern, worldwide, to prevent the cases of HPV infections including precancerous lesions, cervical cancers, and genital warts especially in adolescent female and male population by launching national programs with international alliances. Currently, available prophylactic and therapeutic vaccines are expensive to be used in developing countries for vaccination programs. The recent progress in immunotherapy, biotechnology, recombinant DNA technology and molecular biology along with alternative and complementary medicinal systems have paved novel ways and valuable opportunities to design and develop effective prophylactic and therapeutic vaccines, drugs and treatment approach to counter HPV effectively. Exploration and more researches on such advances could result in the gradual reduction in the incidences of HPV cases across the world. The present review presents a current global scenario and futuristic prospects of the advanced prophylactic and therapeutic approaches against HPV along with recent patents coverage of the progress and advances in drugs, vaccines and therapeutic regimens to effectively combat HPV infections and its cancerous conditions.

## Introduction

Human papilloma virus (HPV) infection is usually a commonly encountered infection (transient) which has attracted the attention of media in recent years due to the advancement in the field of vaccine development and changes in recommendations for screening of cancer (1, 2). HPV is found to be the causative agent for dermatologic diseases and sexually transmitted diseases (3). Across the globe, HPV vaccination has become a public health priority, with many national programs with international alliances launched in several countries of the world. This vaccine can help prevent cases of HPV infection that may result in precancerous lesions, cervical cancers, and genital warts, especially in adolescent females and males. Universal HPV vaccination worldwide will result in a gradual reduction in the incidence of HPV cases (4). The *Papillomaviridae* family comprises more than 130 HPV genotypes that have been isolated from various human neoplasias such as warts, cancers, and cases of recurrent respiratory papillomatosis (RRP) (5). The prevalence and distribution of HPV types differ by geographic region. Moreover, HPV types in 30,848 invasive cervical cancers worldwide revealed differences by geographical region and histological type (6). This virus is a significant cause of mortality and morbidity in the developing countries (7). Globally, cervical cancer ranks as the second most common cancer in women and is responsible for a significant number of deaths (453 million). High-risk HPV, consisting of more than 100 types of HPV, is an important cause of cervical cancer (8, 9). Through the process of immunization along with other therapies, it is possible to control the HPV-associated cancers and such opportunity is created through the process of identifying HPV as a causative agent for malignancies. Against infectious diseases as a tradition use of vaccines have been done as a preventive measure. Success has been achieved in developing prophylactic vaccines against HPV types that cause disease by making the viral L1 (major capsid protein) as target (10, 11). It is, however, to be kept in mind that there is not enough evidence for demonstrating the efficacy of the prophylactic vaccines for treatment of HPV infections and lesions associated with HPV (12, 13).

To control HPV infection, HPV vaccines have been introduced into national immunization programs (14). Since 2013, HPV vaccines (bivalent and quadrivalent) have been included in the national immunization programs of at least 66 nations, including NorthAmerica and Western Europe, primarily (15). Recombinant HPV virus-like particles (VLPs) are being produced at commercial level via heterologous expression of the major capsid protein L1 in yeast or insect cells (16). From the morphology viewpoint, VLPs are similar to natural HPV virions with considerable potentialities to induce animal and human type-specific antibody responses (17). A patent has been granted to (18) for developing a technique of disassembly and reassembly of VLPs to enhance the stability of VLPs. The present HPV vaccines are capable of preventing persistent HPV infections, as well as protecting against premalignant cervical lesions (19). Once HPV infection was established as the main cause of cervical and other types of cancer, our focus turned to typing of HPV by field testing with new diagnostics and applying effective HPV immunization strategies for cancer control (20). Licensed VLP-HPV vaccines should provide long-term safety and effective protection against targeted HPV types. Also, to determine the optimal ages for effective vaccination, their design should address the effects of vaccine modulators, mode of delivery, expandable coverage of HPV types, and effects on males and targeted pre-adolescents or adults (21). HPV vaccines are recommended for both adolescent males and females, and after these vaccines were introduced, a marked reduction in the prevalence of HPV-associated cancers has been observed (22). It is to be noted that no serious adverse events in relation to vaccine has been reported (23, 24).

Among effective prophylactic HPV vaccines, Cervarix (a bivalent vaccine) is protective against adenocarcinoma-causing types including HPV-16, 18, 31, 33 and 45 for a documented period of 6.4 years. Whereas, Gardasil (a quadrivalent vaccine) has been confirmed to protect against genital warts, respiratory papillomatosis, and certain types that cause squamous cell cancer, including HPV-6, 11, 16, 18, and 31, for 5 years (10). Despite these successes, vaccination against HPV has triggered much debate. Also, use of these vaccines in developing countries is essentially non-existent, mainly because of its high cost and the difficulty of introducing them into vaccination programs, as they both require three injections over a 6-month period for girls (25). Gardasil and Cervarix are effective vaccines against HPV, generally, but their coverage of HPV types is limited, and their use in pregnant women is not advised; hence, vaccines with a broader preventative and therapeutic spectrum and better safety profile are desired.

Chimeric vaccines, multivalent vaccines to address HPV types (broader spectrum) or to combine HPV with other pathogens, edible vaccines, biodegradable and mucoadhesive polymer-based vaccines, and various viral vectors harboring recombinant HPV DNA vaccines are being developed for greater immunogenicity than that of current vaccines. Similarly, for a more recipient-friendly vaccine, needleless immunization technologies, including the use of a jet gun, gene gun, and microneedles are being evaluated for HPV cancer prevention (26). Lyophilized HPV pseudovirions have been delivered to murine models using microneedles (27). Studies exploring PubMed, EMBASE, Cochrane Library, and clinicaltrials.gov databases for published reports on immunogenicity and safety of bivalent and quadrivalent vaccines, specifically in Asian populations, have suggested strategies for developing vaccines that elicit enhanced HPV-16- and HPV-18- specific antibody levels (28). In light of the serious consequences of HPV-associated cancers and warts, HPV vaccination should be included in the standard childhood vaccination regimen (29).

The present review presents a current global scenario and futuristic prospects of the advanced prophylactic and therapeutic approaches against HPV along with recent patents coverage of the progress and advances in drugs, vaccines and therapeutic regimens to effectively combat HPV infections and its induced- pathological sequels.

## HPV disease

HPVs belong to a large family of non-enveloped, small, approximately 7.9–8 kbp circular dsDNA viruses that are surrounded by an icosahedral protein capsid composed primarily of a highly immunogenic L1 protein, with a minor contribution from the L2 protein (30, 31). The virus is the cause of squamous epithelial cell proliferation, or common warts, on areas of the body such as the hands, feet, anus, cervix, scrotum, groin, thigh, or penis (32). The life cycle of the virus is divided into five phases. Infection and subsequent uncoating is the first phase wherein there is an affection of the basal cells. Genomic maintenance is the second phase during which there is an expression of the early proteins of the virus, viz., E1 and E2. This is followed by the proliferative phase wherein there is an expression of some other early proteins like E6 and E7. The cell cycle progresses after being stimulated by such proteins. Subsequently, there is a synthesis of the virus which is the fourth phase during which there is an expression of the late viral proteins, viz., L1 and L2. The virus is packaged in the epithelial layer under the influence of these late/structural proteins. During shedding of the dead cells at the stratified epithelial layer, there is the release of the virus (fifth phase) which can then cause infection of other cells (33, 34).

HPVs with different oncogenic potential have been assigned to three main groups: high-risk types, intermediate-risk types, and low-risk types. High-risk types include HPV-16, 18, 31, 33, 35, 39, 45, 51, 52, 56, 58, 59, 68, 73, and 82; intermediate-risk types include HPV-26, 53, and 66; and low risk-types include HPV-6, 11, 40, 42, 43, 44, 54, 61, 70, 72, and 81. Among these types, 70% of cervical cancers result from type 16 and 18 infections, and if multiple HPV types infect women, persistent HPV infections can be established, and cervical cancer exacerbated (26, 35). There is also the involvement of HPV- 33 and 45 in causing squamous cell carcinoma and adenocarcinoma, respectively (36). It is to be mentioned in this regard that the low-risk HPV viruses and the oncogenic types are responsible for causing warts (anogenital) and cervical dysplasia, respectively (34).

HPV is held responsible for the trends in the increase in the rate of oropharyngeal carcinoma as is revealed by molecular detection methods. The viral nucleic acid is detectable in a clinical specimen by polymerase chain reaction at a much greater frequency after the year 2000 as compared to before 1990 in the United States alone. The involvement of HPV in oropharyngeal carcinoma has greatly increased during a span of 20 years in the Netherlands from 1990 to 2010. Similar is the situation in another European country like Spain (37, 38).

Despite this, most HPV infections induce no or only mild cytological abnormalities; and few HPV infections lead to cervical cancer. HPV can also cause cervical and other cancers such as cancer of the vulva, vagina, penis, or anus. It can also cause cancer in the back of the throat, including the base of the tongue and tonsils (39). Usually HPV infections clear within 1–2 years' time, but if multiple HPV types infect a person, the viruses may persist, leading to lethal cancers in various parts of the body. In one study by the U.S. Centers for Disease Control (CDC) conducted from 2004 to 2008, an average of 33,369 HPV-associated cancers was diagnosed annually. Therefore, CDC projected that every year roughly 26,000 new cancer cases occurred as a result of HPV infection, including 18,000 cases in females and 8,000 in males. In the U.S., cervical cancer prevention is based on two approaches: one primary and one secondary. The primary strategy is regular HPV immunizations, and the second strategy is screening of populations at risk or immunized to reduce HPV-related cancers (40).

Among oncogenic HPVs, genotypes 6 and 11 cause laryngeal papillomas and most of the genital warts, whereas types 16 and 18 are responsible for 70% of cervical cancers. To prevent infection with highly transmissible HPV, a quadrivalent (types 6, 11, 16, and 18) HPV vaccine has been evaluated and shown good results in clinical trials. Similarly, a bivalent vaccine (types 16 and 18) has shown greater than 90% protection against persistent HPV infections, even 5 years post-vaccination. In both, the vaccines were non-infectious, DNA-free VLPs, developed using recombinant technology, administered in conjunction with appropriate adjuvants, and three doses at 6-month intervals have been shown to elicit high-titer serum antibodies (41).

A study performed in young women 15–25 years of age to assess the immunogenicity and efficacy of a human papillomavirus 16 and 18 (HPV-16/18) AS04-adjuvanted vaccine against cervical intraepithelial neoplasia (CIN) grade 1 or greater (CIN1+, CIN2+, or CIN3+) showed that vaccination of adolescent girls before their first sexual encounter resulted in better protection and prevention of HPV-associated cervical cancers and persistent infections (42). The HPV-16/18AS04-adjuvanted vaccine reduced the incidence of high-risk human papilloma viruses in a randomized cluster trial in adolescent girls and boys, irrespective of their lifestyles and sexual behavioral patterns (43).

The clinical efficacy of bivalent (HPV-16 and 18), quadrivalent (HPV- 6, 11, 16, and 18), and nonavalent (9vHPV; HPV- 6, 11, 16, 18, 31, 33, 45, 52, and 58) vaccines against cervical cancer was tested. The bivalent and quadrivalent vaccines reduction in the rate of HPV-16 and 18 prevalences significantly compared to that of the 9vHPV vaccine and all three vaccines decreased the morbidity and mortality from cervical cancers resulting from oncogenic HPVs (44). The HPV-16/18-AS04-adjuvanted vaccine, when administered in a one-dose schedule to adolescent girls 9–14 years of age and evaluated for immunogenicity and safety, showed clinically acceptable results compared to a three-dose vaccine schedule for women aged 15–25 years (45). Another clinical trial (controlled phase II/III) that evaluated an HPV-16/18-AS04-adjuvanted vaccine in women showed that the vaccine efficiently reduced HPV-16/18 infection, provided cross-protection against some non-vaccine-type oncogenic HPVs that cause genital warts, and protected against oral, vulvar, and anal HPV infection regardless of the age, geographical location, or sexual practice of study participants (46). One 7-year follow-up study on phase III, double-blind, randomized controlled trial from 2006 to 2014 evaluated the efficacy, safety, and immunogenicity of the HPV-16/18-AS04-adjuvanted vaccine in women older than 25 years (in groups ranging 26–35 years, 36–45 years, and ≥46 years). Control women received aluminum hydroxide, whereas vaccinated women were immunized with the HPV-16/18 vaccine. Results showed that the HPV-16/18 vaccine was effective in all age groups and protected against HPV-associated lesions (CIN1+), and corresponding cytological abnormalities, irrespective of the infecting HPV type. Hence, this vaccine was shown to be reliably effective against HPV infections (47). Studies have shown that immunizations with the HPV-16/18-AS04-adjuvanted vaccine do not promote autoimmune disease (48).

Chronic local inflammation, alone or as a result of oral HPV infection, may play an important role in the etiology of head and neck squamous cell carcinomas (49). A study conducted in over 624 nursing students in Izmir, Turkey, revealed that the students had a very high level of knowledge as far as the risk factors and the transmission modes of the disease are concerned but such knowledge has not been used practically to make the vaccination against HPV successful (50). A mathematical model-based analysis has been utilized for systematic screening in women in the U.S. to protect the health benefits and harms; costs involved in vaccination with the bivalent, quadrivalent or nonavalent vaccine (51). Increased awareness of the health risks of HPV infection, such as cervical cancer and warts that results from education and self-testing is an important component in screening and diagnosing initial infections and advanced cases of HPV-associated cancers (52).

### Advances in developing prophylactic and therapeutic vaccines against HPV

Because the cultivation and propagation of HPV in cell/tissue culture are difficult, developing inactivated or live attenuated HPV vaccines is not a common practice. Therapeutic vaccines against HPV can be categorized into nanoparticle-, bacterial-, live vector- (bacterial and viral), nucleic acid- (DNA and RNA), protein-, peptide-, cell- (cytokine-transduced autologous tumor cells and dendritic cells [DCs]) based vaccines [reviewed in (53) and (54)]. Cytotoxic T lymphocyte (CTL) responses elicited by therapeutic vaccines against HPV early viral gene products E1, E2, E5, E6, and E7 (53). Therapeutic vaccines targeting E6 and E7 (early proteins encoded by HPV) are most common because these proteins are produced in all HPV-infected cells and are vital for cancer cells (53, 55). Therefore, researchers have focused on developing unconventional new-generation prophylactic and therapeutic HPV vaccines targeting capsid proteins or the genome by genetic engineering and recombinant DNA technology. The prophylactic vaccines are relatively safer (56–58). It is interesting to note that there is enhancement of cell mediated immunity by T cell based vaccines in contrast to prophylactic vaccines (that help in generating neutralizing antibodies) (59). At present, the questions that arise regarding vaccination against HPV is the protection period (specially, in relation to cross protection). Moreover the shortcomings of the VLP vaccines, viz., less thermal stability, lack of efficacy therapeutically, cost, etc., are also required to be overcome (60).

Various new approaches are presented as below:

#### Protein-based subunit vaccines

These include subunit or subvirion products that induce protective immunity. Fusion protein PD-E7 vaccines comprise a mutated HPV-16 E7 protein linked to the first 108 amino acids of *Haemophilus influenzae* protein D (PD), adjuvanted with AS02B. Vaccinated patients with oncogenic HPV-positive CINs mount significant E7- and PD-specific IgG responses (61). The therapeutic SGN-00101 vaccine (also known as HspE7, developed by StressGen), based on the fusion of the E7 protein of HPV16 and recombinant heat shock protein 65 (Hsp65) of *Mycobacterium bovis*, was evaluated for protection against anal neoplasia (62). Vaccination with SGN-00101 at a dose of 500 μg administered at 3-week intervals induced immune responses and lesion regression in women with high-grade CINs (63).

##### SGN-00101

as a potential treatment for cervical tumors (64) and RRP (65) was evaluated. SGN-00101 induces the activity of CTLs in women having cervical intraepithelial lesions (CIN) (66). An HPV-6 L2/E7 fusion protein is another protein-based vaccine that induces antibodies against HPV-16 oncoproteins. ProCervix (GTL001) is adjuvanted with Aldara developed by Genticel; it consists of HPV-16 E7/HPV-18 E7 bivalent adenylate cyclase (CyaA)-based vaccine that targets HPV-16 and 18 infections (67). ProCervix has been proposed to clear HPV-16 infection while protecting against later infection with HPV-18. A phase 1 clinical trial for safety and immunogenicity of the ProCervix vaccine revealed that HPV clearance was several-fold higher in the group treated with ProCervix than in the placebo group (68).

#### Peptide-based vaccines

As far as such type of vaccine is concerned, characterization of various specific epitopes has been done for the human MHC class I (HLA-A2) peptides. In mammalian models, the cell-mediated immune response is generated by immunizing with a peptide that carries E6 or E7 origin epitopes (69). Various peptide-based vaccines include extended epitope-specific peptides, synthetic long peptides (SLPs), or lipopeptides. SLPs consisting of peptides from HPV-16 (nine E6 and four E7) (HPV-16-SLP adjuvanted with Montanide ISA-51) were tested in phase II clinical trial (70) and interestingly in case of humans multiple clinical trials have been conducted for studying the HPV-16-SLP (long peptide vaccine) (70–72). Examples of this kind of vaccine include those constructed with HPV-16 E7 12–20, E7 86–93, E7 86–93, E7 11–20, and E7 86–93 lipopeptides (54, 73, 74), HPV-16 E7 86–93 (CIGB-228 vaccine) adjuvanted with very small-sized proteoliposomes (VSSPs) (73), as well as HPV-16 E7 49–57 with a poly-IC (a Toll-like receptor [TLR]3 agonist) and anti-CD40 monoclonal antibody as the TriVax vaccine (75).

#### Epitope-based vaccines

Vaccines designed using a traditional approach involve attenuation of a pathogen by sub-culturing, which is a long and tedious process that can take up to 15 years, and the safety of these vaccines is a matter of concern (76, 77). Bioinformatics is an important multidisciplinary tool that may allow optimization of the health benefits of vaccines.

*In silico* tools, paired with improvements in recombinant DNA (rDNA) technology and knowledge of the host immune response and genetic background of the pathogen, will contribute to the future development of new vaccines (78). The first step toward applying bioinformatics to vaccine development consists of identifying epitopes that are potentially immunoprotective from those that are not (76). Prediction of T and B cell epitopes has been the main focus of immunoinformatics, and over the years many different tools have been developed (79). With the advent of bioinformatics and high-throughput technologies, vaccine research has entered a new era, and vaccine design has benefitted from the development of vaccine databases and *in silico* vaccine design tools (80).

A prediction for the 16 major epitope variants (V1–V16) in the full-length L1 protein of HPV-16 and evaluation of the immunogenicity of these variants and reference DNA vaccine constructs was recently reported by Kumar et al. (81). The results of this study showed that the L500F (V16) and T379P (V8) variants induced a ~2.7-fold (*p* < 0.002) increase and ~0.4-fold (*p* < 0.328) decrease in antibody titer, respectively, after the final injection. This study offered a roadmap for the use of both *in silico* tools and experimental methods to develop DNA-based vaccines. The authors also suggested that multi-epitope DNA vaccines might induce more effective immune response against HPVs with different epitope variants than those constructed without a consideration of this variation (81).

In another study, *in silico* tools (B-cell and T-cell epitope prediction methods) were used to design a subunit vaccine against HPV (82). Using a conserved sequence in the L1 binding protein gene from 20 different sequences, the authors proposed a possible HPV vaccine target. Based on their analysis, the authors reported that the L1_41_ protein of HPV was a promising candidate for vaccine design.

Yao et al. (83) identified E6 and E7 proteins as ideal candidates for therapeutic vaccines against HPV-16 infection. A total of 81 CTL epitopes in HPV-16 E6 (*n* = 59) and E7 (*n* = 22) were predicted using Immune Epitope Database Analysis Resource. Among the 20 clusters of epitopes in HPV-16 E6 generated, cluster 3 contained the most epitopes (10 epitopes), representing HLA-A^*^31:01 and -A^*^33:03. Of the 10 clusters of HPV-16 E7, cluster 3 contained the most epitopes (5 epitopes), representing HLA-A^*^01:01 and -A^*^26:01. Based on their observations, the authors suggested that a cocktail of E6 and E7 epitopes such as 52FAFRDLCIVYR62 of E6 (HLA-A^*^02:06, HLA-A^*^31:01, and HLA-A^*^33:03), 66PYAVCDKCLKF76 of E6 (HLA-A^*^11:01 and HLA-A^*^24:02), 2HGDTPTLHEY11 of E7 (HLA-A^*^01:01 and HLA-A^*^26:01), and 11YMLDLQPETT20 of E7 (HLA-A^*^02:01) could be used to vaccinate more than 50% of all individuals worldwide (83).

de Oliveira et al. (84) reported the designing of a multi-epitope recombinant protein. It contains the immunogenic epitopes of E6 and E7 proteins. This particular recombinant protein can protect against HPV-induced tumors in CD4^+^ T cell-deficient mice but not in mice deficient in CD8^+^ T cells. Moreover, the activities of the T cells that are E6/E7 specific are also enhanced. So the use of this multi-epitope protein is assumed to be a promising approach to design a potent vaccine therapeutically against HPV-induced tumors.

In another study, biopsies of nine cervical cancers (HPV-16-infected) in patients with HLA-A^*^02 were obtained. The E7 oncogene-coding region was reported to be conserved in all tumors. Of the nine samples, the E7_11−19_ peptide (11YMLDLQPET19) was detected by MS3 analysis of the HLA-A^*^02 immunoprecipitate from seven of them. However, of the 13 epitopes predicted using the *in silico* approach, only one was observed through an exquisitely sensitive physical detection method, suggesting that bioinformatic prediction should be used to identify probable epitopes for confirmation by physical detection. Because the conserved E7_11−19_ peptide is the dominant HLA-A^*^02 binding tumor antigen in HPV-16 transformed cervical squamous cells cancers and adenocarcinomas, it has the potential to be used for the development of therapeutic cancer vaccines (85).

Sequence alignment studies have detected that the L2 proteins of HPV are having greater intertype variability than L1 which is responsible for the lower consistency of L2 protein as far as positioning in the variable region is concerned. There is the presence of greater number of epitopes in the region of L1 that is conserved and such regions are the prime targets for antibodies (neutralizing types). It can, therefore, be mentioned without a doubt that as far as designing vaccine is concerned a better target is L1 protein compared to L2 (86). *In silico* approaches to predict the epitopes are highly valuable compared to conventional procedures that are costly and time-consuming. The predicted peptides can then be tested both *in vitro* and *in vivo* to verify their effectiveness in triggering an immune response. Assays with short peptides (overlapping) covering the entire sequence of the targeted protein/antigen are used to define CTL epitopes (MHC Class I restricted). Determination of the epitopes of HPV-16 is also done in the same way (53).

#### Recombinant vaccines

##### Recombinant adeno-associated virus (rAAV)

Intranasal immunization against HPV-16 with recombinant adeno-associated virus (rAAV) type 5 encoding the major capsid protein L1 of HPV-16 showed that a single-dose of this vaccine without an adjuvant was sufficient to elicit high titers of mucosal antibodies in vaginal washes and L1-specific serum antibodies (87). Immunization of mice with a single dose of AAV5-HPV-16 L1 intranasally results in the development of both cell-mediated as well as humoral immunity for a prolonged period (88). Another study showed that rAAV-type 9 administered intranasally to mice induced high-titer and long-lasting neutralizing antibodies against HPV-16 (89). A mutated HPV-16 E6/E7 gene, whose product is not oncogenic, was cloned into an adenoviral vector, and the vector elicited an immune response that increased the clearance of established HPV–positive-cancer *in vivo* (90).

##### Recombinant measles virus (MV) vaccine

A vaccine expressing the L1 capsid protein of HPV-16 that was designed to protect against HPV-16 infection revealed measles virus for to be a valuable vehicle for the development of inexpensive and effective vaccines (91, 92). In another study, Gupta et al. (93) administered a recombinant live-attenuated MV Edmonston-Zagreb (rMVEZ) strain as a viral vector carrying heterologous genes that encoded the L1 major capsid proteins of HPV-16 and HPV-18 to rhesus monkeys (93). HPV-16L1/18L1-specific total IgG antibodies, neutralizing antibodies, and related cellular immune responses in non-human primates were comparable to those in response to the classical recombinant *Pichia pastoris* expressing HPV protein. A patent has been granted to Mendiretta et al. (94) for developing a dual vaccine applicable for treating measles and HPV by using measles vector and inserted genes coding for HPV antigens.

##### Vaccinia virus Ankara (MVA)-based vaccines

The growth of human tumors was effectively controlled by immunization with the recombinant vaccinia virus Ankara (MVA) expressing HPV E2 (MVA-E2). This vaccine showed potential to be used as a therapeutic vaccine (95). The viral vector-based MVA-E2 therapeutic vaccine inhibited HPV growth in high-grade lesions (CIN2 and CIN3) (96). Direct injection of the vaccine has been done in the uterine cervix (97). Immunization with this vaccine can eliminate precancerous lesions (CIN1, CIN2, and CIN3) associated with infection by oncogenic HPV types (98). A phase I/II study of the therapeutic MVA-E2 vaccine indicated that this vaccine is highly effective in inducing immune responses against human papilloma viruses and regression of flat condyloma lesions in men (99). The therapeutic antigen (TA)-HPV vaccine (improved by Celtic Pharmaca, previously Xenova or Cantab) is based on a live recombinant vaccinia virus strain (Wyeth) expressing a modified E6/E7 fusion protein of HPV-16 and HPV-18 (100). Safety and immunogenicity of the vaccine were confirmed in a proportion of those patients vaccinated (101). Injection of TA-HPV vaccine into the deltoid muscle has been done in a clinical study to patients having grade III vulvar intraepithelial neoplasia (VIN) and grade II vaginal intraepithelial neoplasia. After vaccination, identification of the enhancement of T-cell-based immunity (HPV E6 as well as E7 specific) has been done by employing interferon-gamma (IFNγ) ELISPOT assay (102). A booster vaccine TA-CIN (HPV-16 L2/E6/E7) in combination with TA-HPV resulted in shrinkage of vulval intraepithelial neoplasias and symptom relief in some patients, with some showing an immune response (103). Multiple clinical trials have been conducted for evaluation of the efficacy as well as safety of MVA-based vaccine targeting the protein E2 (55, 104). For treating ano-genital lesions (intra-epithelial, including urethral condyloma or anal lesions) induced by HPV, phase III clinical study has been carried out (105). The sequences of attenuated MVA (recombinant) that encodes E6/E7 of HPV-16 (modified) are included in TG4001 vaccine. Further it is to be mentioned that this vaccine is nothing but MVATG8042 suspension (106).

#### Bacteria-based vaccines

**Figure 1:**
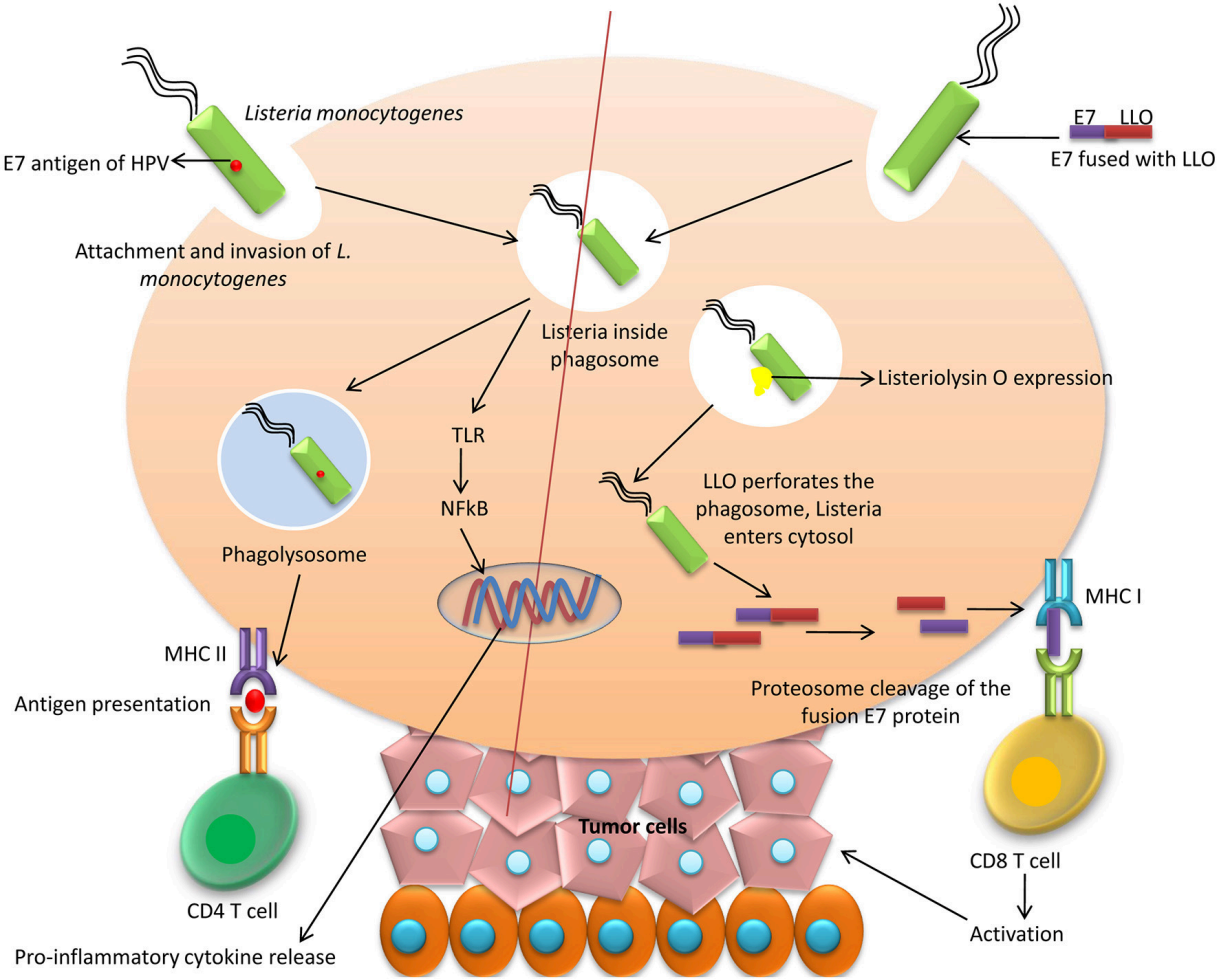
Bacteria-based vaccines. *Listeria monocytogenes* can be used to secrete HPV E7 which activates CD4 helper T cells through MHC II antigen presentation mechanism. Another method of E7 delivery is to fuse E7 to non-hemolytic listeriolysin O (LLO) protein of the bacteria (Lm-LLO-E7) and thereby on delivery causes perforation of phagolysosome due to LLO and E7 protein made available to MHC I which activates cytotoxic T cells.

The reporting of Listeria-based vaccine for therapeutic purpose against HPV was done for the first time in the year 2009 (107). Two different vectors of a live-attenuated *Listeria monocytogenes* (Lm) were engineered by Gunn et al. (108). In one, the Lm vector was secreted with E7 as Lm-E7, and, in the other, E7 was fused to non-hemolytic listeriolysin O (LLO) protein of the bacteria (Lm-LLO-E7) (Figure 1). The most important vaccine with anti-cancer activities is ADXS 11-001 (or ADXS-HPV, formerly known as Lovaxin-C), a therapeutic *Listeria*-based vaccine targeting an HPV E7 antigen (108, 109) and resulting a TNF-alpha (TNF-α) response and IL-2 production by DCs (110). The phase II FAWCETT trial (NCT02399813) is assessing the safety of ADXS11-001 in patients with metastatic squamous cell carcinomas of the anal canal (SCCA) and will be testing for protection against cervical, oropharyngeal, and anal cancers (111). Another bacteria-based vaccine expressing the viral E7 protein has been designed utilizing *Lactobacillus casei* as a vector. The safety of the *L. casei*-based vaccine is relatively high and oral administration is found to be fruitful. For evaluation of the cellular immunity, this particular vaccine has been administered in patients having CIN3. Majority of the patients that have received the vaccine responded well with regression of the disease which is associated with E7 specific cellular immunity. Enzyme-linked immunospot (ELISPOT) assay has been employed for evaluation of the E7 specific-cellular immunity. When treatment with four-six capsules/day is done, there is exhibition of HPV E7 specific-cellular immunity in lymphocytes of the cervix (112, 113).

#### Yeast-based vaccine

A recombinant HPV-16 L1-expressing *Schizosaccharomyces pombe* yeast strain (HPV-16L1 yeast) produced edible HPV vaccines that were administered to female BALB/c mice (114). A codon-optimized HPV-16 L1 gene cloned into a non-integrative expression vector was transformed in *Pichia pastoris* yeast cells. Heparin-Sepharose chromatography was employed to purify L1 protein from the yeast extract. The resulting protein contained native conformational epitopes, as evidenced by immuno-electron microscopy, and showed great potential for use as a low-cost vaccine (115, 116).

#### VLP-based vaccines

There has been the adoption of VLPs as platforms for developing various candidates of HPV second-generation vaccine. VLPs have close resemblance with HPV particles (native) and include conformational epitopes which help in induction of neutralizing antibodies (26). Vaccines based on VLPs are safer to use while offering a display of B-cell epitope at greater density and presentation of T-cell epitopes intracellularly (117). So VLPs are known to be highly immunogenic. Due to the lack of viral gene, the VLPs are fully non-infectious as well as non-oncogenic in nature forming structure resembling the HPV virus outer shell. They induce production of antibodies that react with the virus (26, 118, 119). Moreover, one interesting feature of VLP vaccine is that each of the vaccine has its adjuvant promoting durable immunity (120). But producing them on a large scale is expensive and difficult (54). Zhou et al. (121) explored VLP technology for the development of an HPV vaccine, which led to the most significant development in HPV vaccines and cervical cancer control (121). They expressed recombinant open reading frame (ORF) proteins L1 and L2 of HPV-16 in cells infected with a recombinant form of Vaccinia virus and explored the production of HPV-like particles, which is useful for biochemical studies and can provide a safe source of material for the development of vaccines. One promising alternative approach to producing recombinant VLP antigens is adding subdominant neutralizing epitope in the L2 protein of the HPV (122). In a 5-year study to assess the prophylactic efficacy of a quadrivalent HPV 6/11/16/18 L1 VLP vaccine in 552 adolescent and young adult females of 16–23 years of age, vaccination reduced the incidence of cervical and genital cancers, precancerous dysplasias, and genital warts, and prevented infections with HPV-6, 11, 16, and 18 (123). When immunologic responses in 1106 young females against HPV types 6, 11, 16, and 18 L1 VLP vaccine were measured, 12- to 26-times higher levels of anti-HPV vaccine-type antibodies and an anamnestic protective immune response was observed, with no adverse side effects (124).

Mutant of *Salmonella enterica* serovar Typhi, i.e., Ty21a engineered to produce VLPs with HPV-16 L1 was administered as a potent live HPV vaccine to simultaneously induce protective immunity against cervical cancer and typhoid fever (125). Live bacteria-based HPV vaccines such as attenuated *Shigella* can be used to produce VLP (126) and to promote potent local and systemic immune responses (127). This is a prophylactic, efficient, and low-cost mucosal vaccine. Heterologous production of HPV-16 L1 protein in *Lactococcus lactis* was demonstrated using two vectors, pCYT, and pSEC, designed for intra- or extracellular expression, respectively (128). The results of a study by Cortes-Perez et al. (128) revealed that the use of recombinant food-grade lactic acid bacteria such as the *L. lactis* for the production of L1-based VLPs in a safe mucosal vector is a promising approach to creating HPV-16 prophylactic vaccines.

Abdoli et al. (129) designed HPV-16 VLPs with L1 protein in *Spodoptera frugiperda* 9 (Sf9) insect cells and suggested administration of recombinant baculovirus containing the HPV-16 L1 gene as a prophylactic vaccine and for diagnostic tests (129). Also, a modified baculovirus-based (MultiBac) approach to producing VLPs for heterologous expression of the HPV L1 protein in insect cells was used by Senger et al. (130). Self-assembly of the L1 protein of HPV-6a into VLPs was demonstrated in both L1- and L1+ L2- coexpressing *Saccharomyces cerevisiae* (131). An alternative HPV antigen to elicit an immune response against HPV is the L1 pentameric subunit or capsomere with conserved neutralizing epitopes (132). Expression of recombinant HPV capsomeres in *Escherichia coli* may substantially reduce manufacturing costs. Studies in animal models have shown that HPV capsomeres alone induce lower antibody titers than those in VLPs (132). Expression of the L1 gene of HPV types 6 and 11; 16 and 18 have been reported in *S. cerevisiae* for producing the HPV4 vaccine that is used for protection against persistent infection caused by HPV (133, 134). Another vaccine similar to HPV4 is HPV2, which is used for prevention of oncogenic HPV (23). In another study, a recombinant major capsid L1 protein of HPV-11 was produced intracellularly at high levels in an expression system based on galactose-inducible *S. cerevisiae* with an HPV-6/11 hybrid gene (135).

Moreover, VLPs of HPV-58 with the L1 protein produced in *S. cerevisiae* elicited antibodies and antigen-specific CD4+ and CD8+ T cell responses without the need for an adjuvant (136). Also, HPV-16 and -18 L1 protein expressed in *E. coli* to produce bivalent VLPs has been demonstrated safe and highly immunogenic as a vaccine candidate in preclinical studies (137). Fusion of HPV L1 to the surface of *Shigella sonnei* autotransporter, i.e., IcsA, introduced a new VLP strategy to improve live attenuated *Shigella-*HPV vaccines for better stability and more effective expression (138). Multivalent VLP vaccines for HPV were introduced by Xu et al. (139) and revealed that HPV-31 L1/L2 VLP-based vaccines induced strong type-specific and cross-reactive antibodies. Moreover, tobacco plant-based L1/L2 chimeras containing hybrid epitope sequences of HPV-16 L1/L2 induced anti-L1 and anti-L2 responses, and the antisera neutralized homologous HPV-16 and heterologous HPV-52 pseudovirions in mice (140). Vaccines targeting the L2 minor capsid antigen revealed particularly strong and long-lasting antibody responses in mice, with a Th2 to Th1 shift in response (141). VLPs displaying HPV L2 peptides for capsid display, adjuvant ability, and fusion with early HPV antigens or TLR agonists are in development to improve upon licensed HPV vaccines (142). These vaccines elicit neutralizing antibodies and can block infection with a wide range of HPV types (143).

Recently, the capacity of AS04-adjuvanted vaccines based on VLP chimeras of L1 and two L2 epitopes to protect against HPV was evaluated, and these chimeric vaccines induced immunity and protected against various types of HPV (HPV-6, 11, 16, 31, 35, 39, 45, 58, and 59 as pseudovirions or quasivirions) in both mouse and rabbit challenge models (144). Also, the use of different antigens such as oncogenic peptides, synthetic peptides, DNA, and bacterial antigens may allow the development of effective prophylactic and therapeutic vaccines that can address all of the issues associated with current vaccines.

Purified L1 capsomeres expressed in *E. coli* represent an economical alternative to (VLP)-based prophylactic vaccines against HPV-16 and 18 (145). Schädlich and colleagues reported that the L1ΔN10 protein was particularly immunologic and that L1 constructs could be administered to produce potent immunogenic responses to capsomeres in bacteria as a potentially inexpensive alternative to VLP-based formulations. Furthermore, in another study, tobacco plants were used to express pentameric capsomeres of a modified HPV-16 L1 (L1-2xCysM) protein to induce immunity against HPV (146). Licensing of subunit vaccine (VLP-based) from the L1 protein (plus adjuvant) has been done already (147). A novel VLP with HPV-16 E5 based on a whole gene as well as long multi-epitope gene version of E5 was designed by Cordeiro and collaborators (148). It is interesting to note that for judgment of subunit vaccines in future, the benchmark should be the HPV VLP vaccines (149).

#### DNA vaccines

The safety of DNA (naked) is relatively higher. They are stable and lucrative due to ease of production and they have got application in sustaining expression of antigen in the cells at greater level (150–152). Additionally the DNA vaccines do not evoke anti-DNA antibodies for which their repeated administration can be done (153). Moreover, DNA vaccines offer several other benefits such as having inherent adjuvant properties that are lacking in a traditional peptide or attenuated-virus vaccines, and they are highly effective in treating HPV infections. In this category, ZYC-101 (developed at Eisai, formerly MGI Pharma and previously known as Zycos Inc.), the precursor of amolimogene bepiplasmid, which was evaluated in phase I clinical trial, is based on a bacterial plasmid (BIOTOPE). It encodes 25-residues of a human MHC class I antigen (HLA-DRα) trafficking peptide (MAISGVPVLGFFIIAVLMSAQESWA) fused to an immunogenic peptide derived from the E7 protein of HPV-16 (82LLMGTLGIVCPIC94) (154). This vaccine is effective against anal and cervical dysplasias, with clinical outcomes consisting of regression of AINs (3/12 PRs), as well as regression of CINs (5/15 CRs) (155). Also, lesions showed the greatest regression in patients <25 years of age. In another study, DNA vaccine candidates with HPV-16 E6, E7, and L1 genes from an Iranian isolate inserted into the mammalian expression vector, pcDNA3 elicited therapeutic CTL responses to HPV-16 E6 and E7 proteins (156). In another study, a DNA vaccine expressing the E7 protein of HPV-16 with a mutation in the L-Y-C-Y-E pRb-binding motif at amino acids 23–25 induced a potent CD8+ T cell immune response, as well as promoted significant anti-tumor effects in mice (157).

Moreover, the E7 oncoprotein of HPV linked to an interferon-inducing 17-kDa protein (ISG15) as an adjuvant elicited IFN-γ responses and cytolytic effector CD8 T-cell responses (158). Recently, women with HPV-16 or 18 infections and normal cervical cytology showed potent immune responses to a GTL001 DNA vaccine with acceptable safety (159). Furthermore, Hsp70 can play a significant role in modified HPV-16 E7 and *Mycobacterium tuberculosis* Hsp70 fusion DNA vaccine and introduce as candidate therapeutic tumor vaccine (160). Another vectored DNA-based vaccine is VGX-3100 (IgE leader-E6/E7 DNA) developed by Inovio Pharmaceuticals, which is administered intramuscularly by electroporation and targets the E6 and E7 proteins of HPV-16 and 18 in CINs 2/3 (161). VGX-3100 is the first therapeutic vaccine to show efficacy against CIN2/3 associated with HPV-16 and 18. Safety, immunogenicity, and efficacy of this vaccine were evaluated in phase I clinical trial that recruited 18 women who were previously treated for cervical lesions. The intramuscular route (into the deltoid muscle) was adopted for administration of the vaccine. This was followed by electroporation (162, 163). Finally, these genotype-specific vaccines are in phase I clinical trials and in demand to resolve HPV infections and neoplasias (54).

However, it must be kept in mind that due to shortage of specificity (cell type) DNA vaccines show low immunogenicity. The DNA further lacks the capability (intrinsic) of amplification or nature of spreading to the cells (*in vivo*) in the surrounding. Nevertheless, there may be enhancement of potency of DNA vaccines (used against cervical cancer induced by HPV) by making DNA or the encoded antigen as the target to antigen presenting cells (APCs) and along with this, modification of the feature of APCs (antigen expressing) can boost immune response induced by the vaccine (153). Furthermore, it is also interesting to note that on employing as immunotherapeutic interventions (stand alone), the DNA-based anticancer vaccines are ineffective which can be explained by the establishment of immunosuppression (either systemic or local) (161, 164–167).

#### Plant-based vaccines

Production of candidate HPV vaccines in plant systems is a promising approach. These vaccines have been shown to be efficient and immunogenic, even though they are in the early stages of development (168, 169). One such vaccine is produced in microalgae that have immunomodulatory properties (170, 171). In one study, a plant codon-optimized version of the HPV-11 L1 major capsid protein coding sequence was synthesized and transformed into tobacco and potato plants, resulting in immunologically functional VLPs. The ingestion of this material activated anti-VLP immune responses in mice (172). Moreover, the L1 major capsid protein gene of HPV-16, with or without nuclear localization signals, was integrated into the *Nicotiana tabacum* cv. Xanthi genome and the proteins were assembled into capsomeres to produce VLPs. Rabbits immunized with small doses of the transgenic plants showed weak anti-HPV-16 L1 immune responses (173). Also, HPV-11 L1 major capsid protein in transgenic *Arabidopsis thaliana* ecotype Columbia and *N. tabacum* cv. Xanthi was evaluated as candidates for a low-cost subunit vaccine. Results indicated that immunization of New Zealand white rabbits with ~50 μg of plant-derived HPV-11 L1 induced a weak immune response to native HPV-11 L1 VLPs, as well as to HPV-11 pseudovirions (174).

Expression of an HPV-16 L2 epitope fused to the N- and C-terminus of the coat protein of potato virus X (PVX CP) in transgenic *N. benthamiana* plants was evaluated by Cerovska et al. (175) and reported immunogenic in mice. In mouse sera, antibodies titers against PVX CP and the L2 epitope (108–120) were measured after vaccine delivery (175). Another study revealed that the HPV-16 L1 protein expressed in tobacco chloroplasts induced the self-assembly of VLPs that were highly immunogenic in mice after intraperitoneal injection (176). A circular dsDNA replicon was constructed by cloning a secreted embryonic alkaline phosphatase (SEAP) reporter gene and promoter into a geminivirus-derived plant expression vector that was co-transfected with vectors expressing L1 and L2 proteins into *N. benthamiana* plants, and an HPV-16 pseudovirus was purified. This pseudovirus was neutralized by antisera against current and candidate HPV vaccines and represents a potential plant-derived vaccine (177). A *N. benthamiana*-derived fusion protein with beta-1,3-1,4-glucanase (LicKM) of *Clostridium thermocellum* elicited a protective response with a yield of 100 mg/kg biomass with 99% purity after metal ion chelation and gel filtration purification (178). Transplastomic plants have been used for expression of mutated L1 gene of HPV. This results in the sole production of capsomeres that are pentameric in nature (179, 180).

#### DC-based vaccines

This promising approach has been used to produce HPV therapeutic vaccines. For example, HPV-16 E6 18–26 or HPV-16 E7 12–20 peptides pulsed on immature DCs showed specific immune effects in women that were protective against advanced cervical cancer (181). In another study, a recombinant adenovirus encoding codon-optimized HPV-16 E6 and E7 proteins linked to DCs induced protective immunity against challenge by TC-1 cancer cells *in vivo* (182). For increasing the efficacy of DC-based vaccines not only antigens, but also novel strategies can also be incorporated into DCs. One classical example is the introduction of the small hairpin (sh) RNA-suppressor of cytokine signaling (SOCS1) into HPV-specific DC vaccine (E7 pulsed) (183). For evaluation of the immunogenicity as well as safety of such vaccines (DC-based), conduction of a dose escalation trial (phase I) has been done in cervical cancer (stage IIa or Ib) patients (184) In case of recurrency of cervical cancer also, phase I clinical trial has been conducted (185).

**Figure 2:**
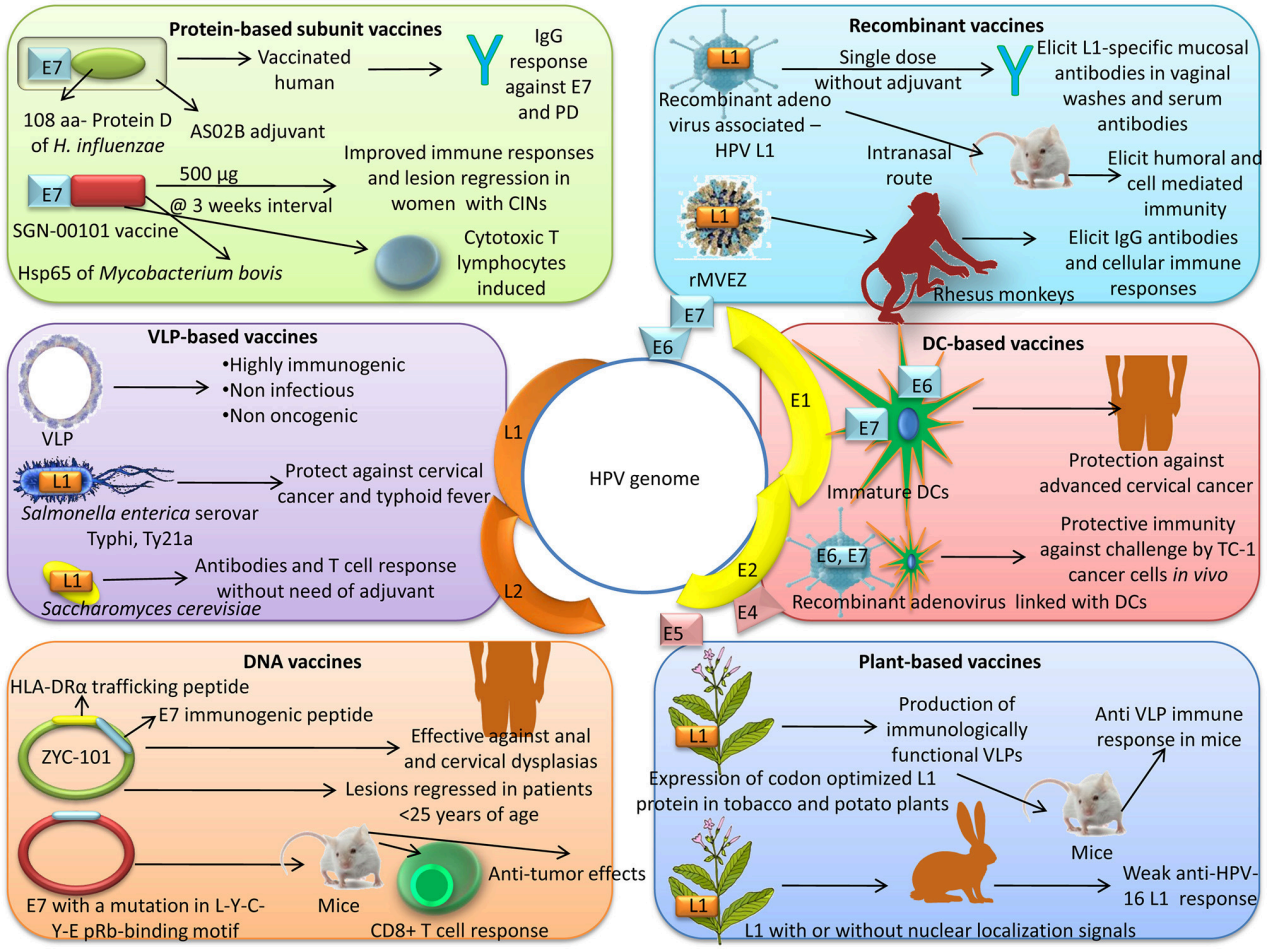
Advanced vaccine technologies available for prevention and control of HPV.

An overview of advanced vaccine technologies available for prevention and control of HPV is depicted in Figure 2.

### Currently available prophylactic vaccines

Currently, there are several licensed prophylactic HPV vaccines, including Cervarix, Gardasil, and Gardasil 9. These vaccines are all based on L1 structural proteins and are designed to inhibit HPV infections on first exposure. Gardasil and Cervarix both contain aluminum-based adjuvants and trigger strong protective immune responses. These two vaccines are beneficial in case of recurrent cervical cancer. Both Gardasil and Cervarix have a similar efficiency against HPV-16 as well as HPV-18 (186–188). They are also safe to used (189). However, case reports from the U.S. Vaccine Adverse Event Reporting System (VAERS) have shown that in spite of their efficacy and safety, a few autoimmune side effects have occurred. These vaccines do not induce sufficient cross-protection against non-vaccine types of HPV, as cross-protective immunity was shown to decline with the time (190, 191). To date, only bivalent and quadrivalent vaccines have shown efficacy; but a newer nonavalent vaccine is also under trial to evaluate its anti-HPV potential (192).

#### Gardasil

A tetravalent vaccine targeting HPV known as Gardasil or Silgard (qHPV-6/11/16/18 vaccine; Kenilworth, NJ, USA) was licensed in 2006 and is composed of 120 μg of antigen per dose (VLP of recombinant L1 HPV-6 [20 μg], VLP of recombinant L1 HPV-11 [40 μg], VLP of recombinant L1 HPV-16 [40 μg], and VLP of recombinant L1 HPV-18 [20 μg]), adjuvanted with 225 μg of aluminum hydroxyphosphate sulfate (123, 124, 193). This vaccine is licensed in many countries and has been shown to be immunogenic and safe and to inhibit infections with other HPV serotypes. Elevated HPV antibodies have been found in all vaccinated persons, ranging from 9 to 45 years of age. Gardasil is administered in 0.5 mL per dose to girls and women ages 9 to 26 years for the control of cervical, vulvar, vaginal, and anal cancers induced by HPV-16 and 18, genital warts (condyloma acuminata) induced by HPV-6 and 11, and dysplastic lesions induced by HPV-6, 11, 16, and 18, as well as grades 1, 2 and 3 CINs, cervical adenocarcinomas *in situ* (AISs), grades 2 and 3 vulvar intraepithelial neoplasias (VINs), grades 2 and 3 vaginal intraepithelial neoplasia (VaINs), and grades 1, 2, and 3 anal intraepithelial neoplasia (AINs). Gardasil is also administered to boys and men 9 to 26 years of age for the control of anal cancers induced by HPV-16 and 18, genital warts (condyloma acuminata) induced by HPV-6 and 11, dysplastic lesions induced by HPV-6, 11, 16, and 18, and grades 1, 2, and 3 AINs.

According to a FUTURE I and FUTURE II analysis, the efficacy of cervical and vulvar neoplasias and grade I VINs are 30, 75, and 48% respectively, and 83% for condyloma acuminate (194). Currently, the duration of protection by Gardasil is considered 9 years (195).

#### Cervarix

A bivalent HPV vaccine (HPV-2; Cervarix, GlaxoSmithKline Biologicals, Rixensart, Belgium) was licensed by the U.S. Food and Drug Administration (FDA) for administration to females 10–25 years of age (196) and has been approved in Europe and Australia (197). This is an L1 VLP vaccine that is produced in a cabbage looper moth cell line (*Trichoplusia ni* [Hi 5]) infected with recombinant baculovirus HPV-16 and 18 L1 and adjuvanted with 500 μg of AS04, which consists of 50 μg of 3-O-deacetylated-4-monophosphoryl lipid A adsorbed into 500 μg of aluminum hydroxide (198). It consists of VLPs of HPV-16 and 18, which induce 70% of cervical cancers worldwide and play important roles in HPV-related vulvar, vaginal, penile, anal, and oropharyngeal cancers (199, 200). The vaccine is composed of 20 μg of recombinant L1 from HPV-16 and 20 μg of recombinant L1 from HPV-18 and is administered intramuscularly in three doses (the second dose given at least 1 month after the initial dose and the third dose given at least 6 months after the initial dose). The AS04 adjuvant in the vaccine induced increased expression of phenotypic maturation markers along with production of pro-inflammatory cytokines as well as cytotoxicity against tumor cells that are positive for HPV when interleukin (IL)-15 dendritic cell (DC) are exposed to the vaccine (201). The vaccine exerted immunity againsy HPV via a novel mode, i.e., boosted innate immunity, including killing of HPV-infected cells by DC and NK cells. The PATRICIA clinical study revealed the efficacy of the vaccine against HPV-16 and 18-associated precancerous cervical lesions to be 92.9–98.1 and 30.4%, respectively, and suggested cross-protection of other oncogenic HPV types such as HPV-31 and 45 (11, 197). The effectiveness of this vaccine against CIN2+ lesions with HPV-16 and 18 has also been reported. The vaccine is well tolerated, highly immunogenic, and capable of generating high titers of neutralizing antibody to HPV-16 and 18 (202, 203). A phase III double-blind, randomized controlled trial of the Cervarix vaccine showed an efficacy of 90.4% against CIN2+ lesions with HPV-16 and 18 (203). Cervarix induces high antibody titers in comparison to natural infection. In women, there has been demonstration of an enhanced humoral immune response (204). Currently, the duration of protection generated by Cervarix is estimated to be 9.4 years (205, 206). One study showed that HPV universal mass vaccination of people in the UK with Cervarix prevented females from developing cervical cancer and protected males from HPV-16 and 18 infections (207).

#### Gardasil 9

Gardasil 9 (Merck and Co., Inc.), a 9vHPV (6/11/16/18/31/33/45/52/58) VLP-based vaccine provides protection against five additional oncogenic types (HPV-31, 33, 45, 52, and 58) and was approved by the FDA on December 10, 2014, for administration to females aged 9–26 years and males aged 9–15 years (208). Gardasil 9 targets up to 90% of genital warts (both Gardasil vaccines also target two HPV types responsible for ~90% of genital warts) (209). A phase III clinical trial in women aged 16–26 years demonstrated the efficacy of the vaccine in inhibiting HPV infection (210). In another study, the safety and efficacy of the 9vHPV vaccine in males and females of 9–26 years were assessed across seven phase III clinical trials. The results showed that this vaccine was well tolerated in subjects with a serious and non-serious adverse event profile similar to that of the Gardasil qHPV vaccine, although injection-site adverse events including pain, swelling, and erythema in both males and females and headache in felames were more common (≥10%) with the 9vHPV vaccine (211). Increased use of the Gardasil 9 vaccine offers the hope of reducing neonatal transmission of HPV and decreasing the incidence and morbidity of recurrent respiratory (laryngeal) pappillomatosis (RRP) (212). A study to estimate the public health effects and cost-effectiveness of vaccination with Gardasil 9 in Germany indicated that immunization of boys with the 9-valent vaccine reduced the incidence of cervical cancer by 24% and anal cancers in males and females of 30, and 14%, respectively, while over a million cases of genital warts would be prevented in 100 years (213). It is important to note that Gardasil-9 has not yet been approved for use in subjects who have received three doses of Gardasil or Cervarix (214). Moreover, Gardasil-9 is expected to be more cost-effective than HPV vaccines currently in use (215, 216).

#### GTL001

For eradication of cells infected with HPV, a therapeutic vaccine (bivalent) known as GTL001 is available. It is a fusion of HPV-16 E7 as well as HPV-18 E7 and detoxified adenylate cyclase (CyaA) of *Bordetella pertussis* binding to specific CD11b+ antigen presenting cells (APC) (217). The interesting feature is there is induction of T cell responses (CD4+ and CD8+) in an antigen specific manner against the viral/tumor antigens. A recombinant CyaA that bears the HPV-16 E7 (antigen) when used for vaccination intradermally causes induction of a T cell response. This requires adjuvantation with a Toll-like receptor 9 agonist, i.e., CpG oligodeoxynucleotide (ODN1826). This ultimately results in elimination of tumors that express HPV-16 E7 (218, 219). There are two GTL001 formulations, viz., a solution form and a powder form (which is more concentrated in nature) adjuvanted with imiquimod cream. The formulations have been tested in a clinical trial (phase I) and both are found to be safe and induced E7-specific CTL responses (159).

#### 9-valent HPV vaccine

The safety as well as efficacy of the 9-valent vaccine providing protection against HPV types 6, 11, 16, 18, 31, 33, 45, 52, and 58 has been proven recently. This will help further in reducing the incidences of infection due to HPV along with the cancer related to the virus. Moreover, a herd immunity is generated for providing indirect protection to individuals that are unvaccinated (220). In boys as well as girls in the age group of 9–15 years, geometric mean titer (GMT) (non-inferior) is generated by 9-valent vaccine (208, 221, 222). The vaccine efficacy is proven also in 16–26 years age group of males (223).

### Safety of the currentluy used HPV vaccines

Safety of HPV vaccines was studied in both clinical trials before they were licensed and through post-licensure surveillance programmes (224–233). Like any other vaccines administered by intramuscular route, the HPV vaccinees may get inflammation (pain, erythema, swelling and pruritus) at the injection site. The other side effects include pyrexia, headache, chill, weakness, malaise, myalgia, and joint pain. These HPV vaccine-related adverse effects (AEs) usually occur from day 1 to day 15 after vaccination and mostly are mild; the vaccines are believed to be well tolerated in girls, boys and young women (234). Nevertheless, serious HPV vaccine related AEs that required hospitalization occurred in vaccinees of 9-valent HPV vaccine including asthmatic attack in a 10-year-old boy who had experienced seasonal allergy and bronchial asthma; high fever (>38°C), body pain, headache, and malaise in a 26-year-old woman; and occipital headache with photophobia, nausia and chill in a 23-year-old woman (234). In a large cohort study of >2 million young girls (aged 13–16 years) between 2008 and 2012 in France of which 37% received HPV vaccine, autoimmune diseases (AID) occurred in 4,096 subjects during the follow-up time (mean of 33 months). The incidence of AID was not increased after HPV vaccination, except Guillain-Barre syndrome (GBS) which was found in 1.4 per 100,000 vaccinees *versus* 0.4 per 100,000 non-vaccinated subjects (232). No increase risk of GBS was observed following HPV vaccination in England (235). A systemic review and meta-analysis of 11 studies did not find any evidence of increased demyelinating diseases after HPV vaccination (233). However, at least 10 cases of neurological events were reported worldwide after HPV vaccination (236–241). A case-control epidemiological study of the vaccine adverse event reporting system (VAERS) database was undertaken to evaluate the risk for reported autoimmune adverse events following quadrivalent HPV vaccination (242). Cases with gastroenteritis, rheumatoid artritis, thrombocytopenia, SLE, vasculitis, alopecia, CNS demyelinating conditions, ovarian damage, or irritable bowel syndrome were significantly more likely than control to have received quadrivalent HPV vaccine (242).

Safety of HPV vaccines in pregnancy or immediately pre-conceptually has been reviewed (230). The HPV vaccination concerns were not only the maternal safety, but also (and more) on the teratogenicity and other fetal adverse events (AEs) following HPV vaccination, including spontaneous miscarriage, preterm birth, congenital malformations, and fetal decease. Pooled results from 11 studies which compared 16,142 women who received at least one dose of either bivalent (2vHPV, Cervarix®) or quadrivalent (4vHPV, Gardasil®) vaccine, with 13,811 girls/women who received control vaccine (hepatitis A vaccine) indicated that the AEs in women who received the HPV vaccine were not greater than the controls, among the age groups 10–14, 15–25, and >25 years and the follow-up periods 0–7, 7–12, and >12 months. For the fetal safety, none of the studies reported a significant increased rate of spontaneous abortion and other fetal outcomes in overall subgroup analyses (e.g., age, interval of time of conception and nearest vaccination, number of vaccinations) compared with controls. The conclusion was the risk of AEs during pregnancy is unrelated to HPV vaccination before or during pregnancy (230).

## Therapeutic approaches to HPV-induced disease

Currently, there are no HPV therapies available. Removal of the abnormal tissue by surgical operation is currently the recommended cure for cervical dysplasia. Removal of the affected tissue, e.g., cervical conizations, however, can lead to premature births. Thus, development of non-invasive treatment therapies for HPV-induced cancers is needed, and therapeutic HPV vaccines are a promising strategy (243, 244).

One study demonstrated the therapeutic potential of curcumin in high-risk HPV-infected oral cancer cells. Curcumin down-regulated HPV transcription by suppressing the cellular transcription factors AP-1 and NF-κB and selectively inhibited E6 oncogene-mediated p53 degradation during carcinogenesis in HPV-16-positive oral cancer cells (245–248). Another study showed that certain cervical cancers did not express HPV oncogenes E6 and E7, and these were considered HPV-inactive tumors because they showed increased WNT/β-catenin and sonic hedgehog signaling, decreased DNA methylation, enriched non-synonymous somatic mutations specifically targeting the TP53, ARID, WNT, and PI3K pathways. Hence, these tumors can be treated by therapies targeting WNT, PI3K, and/or TP53 mutations (249).

The regulatory activity of the immune system is influenced by either chemotherapy or radiotherapy and in tumor models of mice in combination with vaccination can increase the efficacy of T cell-based immunity against HPV infection (250). The lesions induced by HPV (that are of lesser risk and shows infestation with regulatory T cells) can be successfully treated by the use of cyclophosphamide at low dose thereby altering the local environment of the immune system. In recent past, properly planned randomized trial has been introduced in patients with cervical cancer that shows metastasis to compare solely chemotherapy versus chemotherapy combined with a long synthetic peptide of HPV-16. Adoptive cell therapy (ACT) or antibody based-therapeutic approaches have been proven to be successful for treatment of patients with melanoma (251, 252). Earlier, it was evident from various sources that effector T cells that are specific to HPV can be obtained with consistency from cervical cancer patients. By the significance of local microenvironment of HPV-induced lesions, there may be a shift in the local balance of immune effectors through treatment. For example, uses of cyclooxygenase 2 (COX2) inhibitors (through inhibition of prostaglandin E2 production) or inhibitors of transforming growth factor beta (TGFβ) receptor kinase (type I), anti-IL-6 or anti-IL-10 antibodies may prove to be efficacious. Ultimately, the main goal is a suppression of regulatory T cells and a generation of an effective effector T cell microenvironment by identifying the combination that is optimum in enhanced trafficking of the immune effector cells and their efficiency at the affected site. This ultimately provides scope to the immunity induced by vaccination for effective eradication of lesions that are persistent. In this regard, a good example is the use of imiquimod for priming the microenvironment to clear successfully HPV induced-vulvar lesions that are immune-mediated (253, 254).

### Photodynamic therapy

For eliminating malignancies at an early Stage as well as for palliative treatment of cutaneous Tumors Along With Tumors of lungs and esophagus (at late stage), photodynamic therapy (PDT) is an option already approved by Food and Drug Administration (FDA) (255–258). PDT is another approach used widely to treat various cancers. Topical administration of PDT is considered to be most appropriate for cervical and vulval intraepithelial lesions (259). 5-Aminolevulinic acid (ALA)-mediated PDT to treat HPV-associated cervical condyloma has been evaluated for HPV-6, 11, 16, and 18. Complete remission was observed after 1–4 treatments in 98.2% of cases, resulting in an HPV clearance rate of 83.9%, with no evidence of cervical structural changes. This indicates the efficacy and safety of the PDT (260). Topical ALA-PDT has been found efficacious and well tolerated when used to treat high-risk HPV infections (259). A photosensitizer dye IR700 coupled with HPV VLPs, when exposed to cervical cancer cell lines and 690-nm light causes necrosis-like cell death by inducing the influx of CD8+ and CD4+ T-cells into the treated tumors (261).

Hexaminolevulinate-mediated PDT has been found to be safe for treating cervical intraepithelial neoplasia (CIN) (262, 263). Immune cell infiltration is a striking feature of photodynamic therapy, and it forms the basis for treatment of neoplasms that are HPV-associated; especially in the case of vulvar intraepithelial neoplasia (VIN). In chronic VIN, there is an alteration of the immunological balance if photodynamic therapy is employed. This leads to clearance of virus as well as lesions (258). Nevertheless for treating HPV infection the effectiveness of antimicrobial photodynamic therapy (APDT) cannot be denied (264, 265).

### Cryotherapy

For treating cervical intraepithelial neoplasia, the efficacy along with safety and acceptability of cryotherapy is well documented. The cure rate with cryotherapy is very high (266). In low as well as middle income countries, cryotherapy seems to be suitable. But the shortage of refrigerant gas creates hindrance in the application of cryotherapy for treating HPV (267, 268). It is interesting to note that within a period of 3 months one-fourth of the infection due to high risk HPV (hrHPV) can be cleared by cryotherapy (269).

### Cytotoxic agents

A few cytotoxic agents, including podophyllin or trichloroacetic acid, have been used topically to remove genital warts (270), while 5-fluorouracil has been used to a lesser extent because it elicits a strong inflammatory reaction (271). Anti-cancer agents, i.e., arsenic trioxide (As_2_O_3_) and carboplatin target transcription factors AP-1 and NF-κB, play important roles in the expression of HPV oncoproteins E6 and E7 (272, 273). Some immunomodulators like imiquimod have records of safety and efficacy in treating HPV-caused genital warts (274). *Aspergillus, Gliocladium*, and *Penicillium* species produce tricyclic alkaloid gliotoxin (275) and effectively reduce the proliferation of HPV-18 infected cells by inducing Bax, caspase-3, caspase-8, and caspase-9 and suppressing Bcl-2 (276).

### Antiviral drugs

Cis-retinoic acid is used as adjunctive therapy for treating HPV-induced lesion of the larynx; but due to efficacy issues, the drug had been discontinued in patients suffering from recurrent respiratory papillomatosis (RRP) (65). At present, the most common drug used in adjuvant therapy of RRP is cidofovir. The drug is used at a concentration of 5 mg/mL with a dose up to the limit of 3 mg/kg. Such concentration and dose regimen can be followed both in children as well as adults (65, 277–279). Cidofovir can also be administered through inhalation, and in the near future such inhalation therapy may provide scope further to perform research on the clinical ground (280). The expression of E6 as well as E7, has been reduced by cidofovir. This drug is also involved in the reduction of metastatic characteristics of tumor cells that are positive for HPV (281). Ribavirin and acyclovir have also been used from time to time, but their clinical efficacy is questionable (65). For reduction of the size of precancerous lesions of cervix (PLC), an essential oil (natural) containing drug known as anti-viral 2 (AV2) has been introduced. The drug contains various organic compounds, viz., geraniol, eugenol, nerolidol, and carvone which can confer a broad spectrum effect (both on oral administration or topical application). More than half of the size of lesion is reduced when AV2 is applied [(282), http://www.cesa-alliance.com/webapp/index.html, (268)]. Xinfuning (a recombinant human interferon α-2b vaginal effervescent capsule), used to treat vaginal infections, enhances NK cell activity (283).

Both *in vitro* as well as *in vivo*, the activity of interferons (IFNs) against HPV is well proven. Due to the anti-viral activity, anti-proliferative nature and capability to generate host immune response, interferons are quiet noteworthy for treatment of HPV (284). For adjuvant therapy against papillomatosis of larynx, interferons are among the earliest agents to be adopted. Biphasic vesicles are used in recent time to deliver IFN-α topically that ensures its delivery locally for a prolonged period without much exposure systemically (285, 286). IFN-α co-administered with retinoids has shown promising results when used to treat cervical carcinomas (287). Combined therapy with IFN-α and ribavirin has been found to be effective against perianal and genital infection caused by HPV (288). Similarly, pegylated interferon along with ribavirin is useful for treating disseminated HPV infection (289). Treatment with IFN-α has been shown efficacious in reducing the rate of condyloma recurrence (284).

### Herbal medicines

In the Chinese herbal medicine system, several plants with anti-HPV activity have been identified. The Chinese medicine named Paiteling, containing folium, sophora, cnidium, gall, and javanica oil, inhibits HPV by destroying mitochondrial and other membranes to cause necrosis (290). Carrageenan isolated from red algae, is known to bind HPV virions and inhibit post-attachment entry (291). Carrageenan gel as a sexual lubricant has shown efficacy in preventing infection with an HPV-16 pseudovirus (292).

Significant activities against HPV are shown by certain Chinese medicines (traditional) which are used for preventing as well as treating cancer in relation to HPV. The inhibitory effect of Chai Hu (from roots of *Bupleurum chinense*) on infection due to HPV is well known. This particular medicine has been found to interfere with DNA expression of HPV in genital warts. Youdujing is another medicine that is responsible for reversion of the cervical lesion function in patients having a greater risk of infection due to HPV (293–295). Inhibition of the risk of infection due to HPV can be done by Paiteling consisting of folium as well as javanica oil as essential components. Moreover, this medicine also contains gall and cnidium as well as sophora all of which can ultimately cause the specific destruction of the mitochondria as well as other biological membranes ultimately resulting in degeneration of cells along with programmed cell death (290). Treating with fraction of *Pinellia* extract causes reduction of the expression of mRNA and level of protein of HPV E6 whereas there is increase in the protein level as well as mRNA of p53 in cancer cells of the cervix. The antitumor effect of the *Pinellia* extract fraction is thought to be due to the down-regulation of expression of HPV E6 gene and p53 gene upregulation (296, 297). There is reduction of viral load along with improvement of cytological as well as pathological results in patients with infected cervix by application of Zibai gel (298). Youdujing cream is clinically effective and for condyloma acuminatum it is a popular choice. The amplification of HPV-DNA is inhibited as is evident by *in vitro* experiments to reveal the therapeutic efficacy of Youdujing in lesions of the genital tract (299–301).

### Ranpirnase RNAse

Ranpirnase RNAse is a peptide that cleaves double-stranded RNA (dsRNA). It can eradicate HPV from cultured cells. This drug has been used to treat several malignancies with few side effects, even with a transiently increased serum creatinine level. Three different formulations containing 1 mg/mL ranpirnase were applied topically to genital or anal warts of male volunteers, and it was moderately tolerated in these patients with a mean healing time 30 days (302). HPV-11 is the HPV type primarily responsible for genital warts, and ranpirnase is effective against this type, as well as HPV-16. In a patent application by Sulley and Squiquera (303), the enzyme is formulated with a vehicle that does not affect its activity and can be applied topically to genital warts for potential approval as a sexual lubricant. A close variant of ranpirnase has been granted with patent, which contained three mutations (I11V, D20B, and S103R) was found non-toxic and well-tolerated in humans (304).

### RNA interference (RNAi)-based therapies

For cancer therapies based on RNAi, an ideal model system is the HPV-induced tumors because there is expression of E6 as well as E7 (the oncogenes responsible for causing cervical cancer) only on tumor cells (305). As far as the RNAi therapy is concerned, any of the non-structural (early genes) or structural genes (late genes) of HPV can be targeted (306). The two siRNAs targeting the E6/E7 promoter and E7 transcripts, and thereby knocking down E6 and E7 mRNAs elicit high levels of TP53 expression. Subsequently, apoptosis is induced in cell lines of cervical cancer origin which are positive for HPV-16 (307–309). Nine siRNAs designed by Chang et al. (310) were found to target E6 or E6/E7 mRNA of HPV-16 and 18 specifically, and intratumoral administration of these siRNAs resulted in induction of cervical cancer cell apoptosis. In mice (immunocompetent), there is development of tumors (small sized) when HPV-16 E7 siRNAs are used for pre-treating tissue culture-1 cells of murine origin (311). In Caski cells, there may be suppression of tumors if siRNAs are injected intratumorally (312). HPV-siRNA plasmids were constructed using a pTOPO-U6 or pTOPO-U6II vector. HPV-16 and 18-type siRNA libraries have been screened for potent siRNAs, which will subsequently be validated in *in vitro* and *in vivo* experiments (313). However in the clinical setting, the success of RNAi-based therapies is limited (314). It is further interesting to note that the cisplastin sensitivity of cancerous cells is increased when siRNA is used to target oncogenes of HPV as it leads to reactivation of p53 pathway (305).

### Localized immunomodulation

For clearance or suppression of infection due to HPV immunologically in a normal manner, the usefulness of localized immunomodulation has already been proven. The Aldara cream contains an active agent called imiquimod (a Toll like receptor-7 agonist) and this cream can be used topically. There is release of interferons (type I) along with proinflammatory cytokines due to activation of macrophages, dendritic cells along with keratinocytes by imiquimod (315, 316). Imiquimod enhances immunity by activating a Th1 response (317). Imiquimod causes side effects that include irritation at the site of application. Green tea leaves contain an extract known as polyphenon E (sinecatechins) that causes immunomodulatory stimulation of the clearance of the virus (318).

### Immunotherapy

Immunotherapy has emerged as an adjunctive treatment for standard cancer treatments (surgery, radiotherapy and/or chemotherapy). Substances used in the cancer immunotherapy include non-specific immune stimulators, cytokines, monoclonal antibodies and adoptive or engineered autologous immune cells, mainly T cells. The same strategies canbe applied for treatment of HPV-induced cancers. Bacillus Calmette-Guerin (BCG) given *via* catheter into bladder with tumor mass in several cycles over several months reduces the otherwise high recurence and progression rates of bladder cancer by causing stimulation of effective immune response against cancer cells (American Cancer Society). Cytokines (such as interferons) enhance immune system aganst cancers. Therapeutic antibodies, nakedly or conjugated (loaded) with radioisotope, toxin, or drug, kill directly the cancer cells. Some antibodies recognizes molecule which is highly expressed on cancer cells such that the cells are better seen by the effector immune system for antibody-dependent cell-mediated cytotoxicity (ADCC) or complement-mediated cell lysis. Anitbody binding to cell surface receptor can also cause inhibition of downstream cell signaling and prevent cancer cell shading of decoy to increase effectiveness of the host immune system. Monoclonal antibodies in the form of bispecific T cell engager (BITE) bind to cancer cell and effector T cell simultaneously and bring them into vinicity for increasing the effector cell effectiveness. Antibodies to immune checkpoint molecules on effector T cells such as programmed cell death-1 (PD1) and cytotoxic lymphocyte antigen-4 (CTLA-4) restores the effector T cell activity which is suppressed by ligands highly expressed on the cancer cells leading consequently to not only cancer cell death, but also death of regulatory T cells in the tumor environment. Anti-PD-1/OX40 monoclonal antibody treatment increased CD4^+^ and CD8^+^ cells and decreased immunosuppressive CD4+FoxP3+ regulatory T (Treg) cells (319). Chimeric antigen receptor T cells (engineered patient's own T cells) (320) and cytokine activated adoptive/autologous tumor infiltrating lymphocytes (321) are effective in several cancer immunotherapy. These options canbe adopted for treatment of HPV-mediated cancers. Two monoclonal antibodies against the L1 protein of HPV-16 have been produced for diagnostic and therapeutic purposes (322). Anti-HPV 16/18 E6 (C1P5; Abcam) administered repeatedly induced apoptosis of HPV-related cervical cancer (323).

### Miscellaneous therapies

Cimetidine has been found to be useful for papillomas of conjunctiva (in case of ocular surface infection due to HPV). It augments the immune system by inhibition of T suppressor cell function and enhances delayed-type hypersensitivity (DTH) (324). One interesting immunomodulator is dinitrochlorobenzene (DNCB) which may cause induction of DTH response; thereby causing regression of tumor. It has got direct application and is useful in case of surgical failure (325). There is reduction in the rate of recurrence of HPV-induced tumors by use of radiation therapy. Additionally chemotherapy eye drops are found to be efficacious in clinical various trials (326). It is also interesting to find that there is effective inhibition of the growth of tumors (expressing HPV E6 as well as E7) by cetuximab when grafting is done in severe combined immunodeficient (SCID) mice (327).

**Figure 3:**
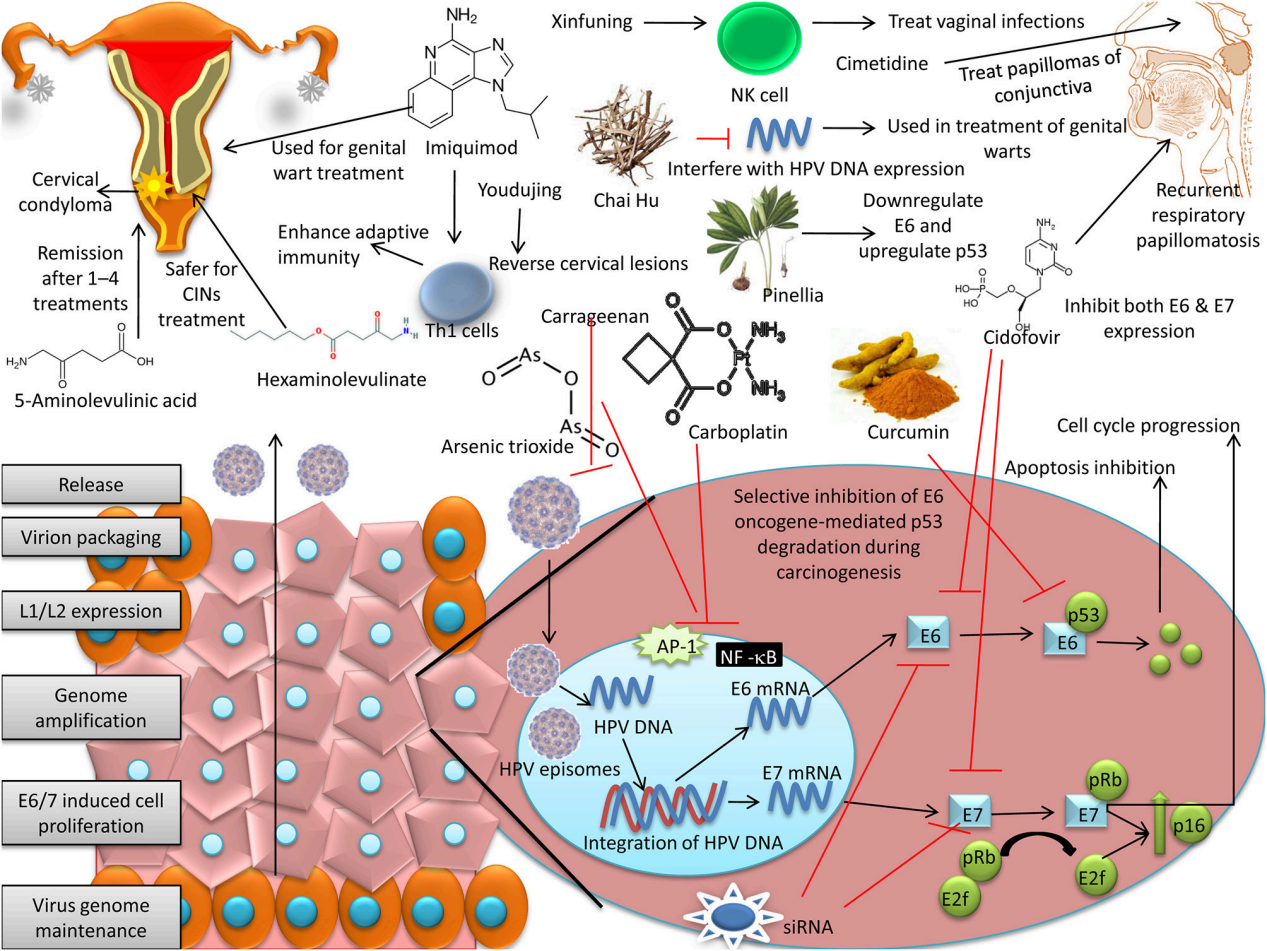
Various therapeutic approaches available for treatment of HPV.

An overview on various therapeutic approaches available for treatment of HPV is depicted in Figure 3. Different patents recital to the HPV are provided in Table 1 and the **v**arious therapies available to treat HPV are summarized in Table 2.

**Table 1:** Different patents recital to the human papilloma virus (HPV).

**S. No**.	**Title of patent**	**Patent No**.	**Inventors**	**Description of patent**	**Publication date**	**Status**	**Reference**
1.	Compounds for the treatment of cancers associated with human papilloma virus	WO 2012176163 A2	Piramal SA, Padigaru M, Agarwal VR, Deshpande GA	Pyrrolidine substituted with flavone derivatives	December 27, 2012	Application	(328)
2.	Compounds for the treatment of HPV-induced carcinoma	WO 2016027005 A1	Rajendran SK, Paul P, Cheng YBF, Eklund P, Eriksson JE	Mixture of pharmaceutically acceptable salts	February 25, 2016	Application	(329)
3.	Methods of treating human papilloma virus	US 8663964 B2	Saxena SK, Ardelt W	Ranpirnase and 805 variant of ranpirnase have anti-viral activity against type 11 HPV	March 4, 2014	Grant	(304)
4.	Topical composition containing ranpirnase	WO 2016028634 A1	Sulley J, Squiquera L	Enzymatically-active ranpirnase with liquid, gel, ointment, or serum as vehicle with no interference with enzymatic activity and to be applied externally	February 25, 2016	Application	(303)
5.	Human papilloma virus E7 antigen compositions and uses thereof	US 20130209402 A1	Webb JR, Wick DA	Use of E7 antigen from two or more HPV types in form of compound	August 15, 2013	Application	(330)
6.	Method of vaccination against human papilloma virus	WO 2013139744 A1	Colau BDA, Giannini S, Lockman L	VLPs with an adjuvant comprising TLR agonist	September 26, 2013	Application	(331)
7.	Recombinant protein carrying human papilloma virus epitopes inserted in an adenylate cyclase protein or fragment	US 8637039 B2	Preville XEE, Leclerc C, Ladant D, Timmerman B	One or several epitopes inserted in permissive sites of an adenylate cyclase (CyaA) protein so as to target antigen presenting cells	January 28, 2014	Grant	(332)
8.	Human papilloma virus treatment	US 20080063661 A1	Neefe J, Goldstone S, Winnett M, Siegel M, Boux L	A composition having a heat shock protein or an immunostimulatory fragment and human papilloma virus antigenic component	March 13, 2008	Application	(333)
9.	Methods of treatment of HPV related diseases	WO 2015021155 A1	Wu TC, Hung CF, Roden R	Mucosal tissue administration of therapeutic HPV vaccines, in a prime-boost regimen	February 12, 2015	Application	(334)
10.	Compositions and methods for cell targeted HPV treatment	WO 2016196282 A1	Quake SR, Wang J	A targetable zinc-finger nuclease, selectively target HPV genome	December 08, 2016	Application	(335)
11.	Use of new type of anti-HPV pharmaceutical preparation	EP 3072512 A1	Liu T, Meng S, Zhao L, Hong G	Combination of paracetamol and a pharmaceutical preparation for prevention and treatment of clinical symptoms caused by HPV infections, including warts, vulvar cancer, penile neoplasms, anal carcinoma, prostate cancer, bladder cancer, cervical cancer, rectal cancer, oral cancer and tonsil cancer	September 28, 2016	Application	(336)
12.	Multitype HPV peptide compositions and methods for treatment or prevention of human papilloma virus infections	US 20100297144 A1	Roden R	Embodiment contains L2 epitope from at least two HPV types	November 25, 2010	Application	(337)
13.	Compound podophyllotoxin gel for treating HPV virus and preparation method thereof	CN 105287365 A	Lide Y, Wensi Z, Qixiong X	The gel includes salicylic acid, phenol, acetoxychavicol acetate and podophyllotoxin in ethanol and afterwords inclusion of glycerin, carbomer 940 and azone	February 03, 2016	Application	(338)
14.	Pharmaceutical composition using *Stryphnodendron* extracts for treating HPV infections	US 9023405 B2	Neto MADFL, Caetano LC, Neto PADSP, Silva ZPD	Administration of an extract of *Abarema cochliocarpos*	May 05, 2015	Grant	(339)
15.	Synergistic antiviral compositions comprising a viral attachment inhibitor, integration inhibitor, and proviral transcription inhibitor	US 9005889 B2	Huang RCC, Abd-Elazem IS	A pharmaceutical composition containing triterpenoid saponin, lithospermic acid and nordihydroguaiaretic acid (NDGA) derivative	April 14, 2015	Grant	(340)
16.	Vaginal cream for the treatment of human papilloma virus infection comprising docosanol, turmeric, amla and aloe	WO 2013171607 A1	Bertin W, Pecora T	For topical treatment of skin lesions and of mucous membranes	November 21, 2013	Application	(341)
17.	Treatment of cancer and benign proliferative disorders	WO 2015059485 A1	Hampson I, Hampson L	Methods to treat HPV associated dysplasia of the cervix	April 30, 2015	Application	(342)
18.	Recombinant human papilloma virus type 18 vaccine	US 5820870 A	Joyce JG, George HA, Hofman KJ, Jansen KU, Neeper MP	Vaccines for human papilloma virus type 18	13 Oct 1998	Grant	(343)
19.	Composition for treatment and preventative of the human papilloma virus infection, ulcerations and boils	CA 2967506 A1	Alessa NAA	Based on herbal composition of a herbal combination, consists of *Aloe vera, Citrullus colocynthis*, hard sea salt, myrrh and garlic	18 Jan 2016	Application	(344)
20.	Human papilloma virus vaccine with disassembled and reassembled virus-like particles	US 6245568 B1	Volkin DB, Heryk Mach H, Shi L	Vaccine formulations containing virus-like particles (VLPs); those are made stable with enhanced shelf-life, by subjecting the VLPs to a disassembly and reassembly process	12 Jun 2001	Grant	(18)
21.	Methods and materials for treating human papilloma virus infections	US 7704965 B2	Clawson GA, Pa WH, Thiboutot D, Christensen N	Antisense oligonucleotides designed to interact with HPV E6/E7 RNAs to reduce the number of HPV infected cells	27 Apr 2010	Grant	(345)
22.	Topical treatment and prevention of human papilloma virus infection	US 20050272700 A1	Buyuktimkin S, Buyuktimkin N, Yeager J	Dietary indole compound-complexed with cyclodextrin to form a stable suspension for topical application	8 Dec 2005	Application	(346)
23.	Guanidinyl-substituted polyamides useful for treating human papilloma virus	US 9133228 B2	Bashkin J, Edwards TG, Fisher C, Harris GD, Jr., Koeller KJ Jr	Polyamide compositions containing guanidinyl radicals	15 Sep 2015	Grant	(347)
24.	Combined measles-human papilloma vaccine	US 9623098 B2	Mendiretta SK, Glueck R, Giannino V, Cantarella G, Scuderi F, Billeter M, Fazzio A	Recombinant measles virus vectors containing heterologous nucleic acid encoding for one or more antigens derived from HPV	18 Apr 2017	Grant	(94)
25.	Genes encoding major capsid protein L1 of human papilloma virus	WO 2009076824 A1	Zhang G, Shen Q, Lei J, Yuan J, Zhang M, Zhang Q, Xiong Y, Wei R, Wu K	Codon-optimized genes encoding major capsid protein L1 of human papilloma virus is expressed in yeast cells and used as an immunogen	25 Jun 2009	Application	(348)
26.	Vaccines for human papilloma virus and methods for using the same	US 9050287 B2	Weiner DB, Yan J	A recombinant vaccine having consensus E6 and E7 genes	9 Jun 2015	Grant	(349)
27.	Human papilloma virus vectors	US 6399383 b1	Apt D, Khavari P, Stemmer W.P.C	This particular invention provides with vectors that are useful in gene therapy against human papilloma virus.	4 Jun 2002	Grant	(350)

**Table 2:** Various therapies available to treat human papillomavirus (HPV).

**S. No**.	**Therapy available**	**Chemical used**	**Size of treatment group**	**Application format/ Modality**	**HPV types surveyed for/ detected**	**Reference(s)**
1.	Photodynamic therapy	5-Aminolevulinic acid	39 patients	10% thermogel	Not specified	(259)
			30 women	Topical 6% 5-ALA in gel form	HPV types 16 and 18	(351)
			41 patients	–	HPV types 11, 6, 1, 56, 52, 45, 53, 16, 31, 35, 39, 59, 51, 58, and 81	(352)
		Hexaminolevulinate (higher bioavailablity than 5-ALA)	24 non-pregnant women	(10 mM) thermogel	HPV types 16, 18, 31, 33, 35, 39, 45, 51, 52, 56, 58, 59, and 68.	(353)
			262 women of childbearing age	5%, 1%, 0.2% ointment	HPVtypes 16, 18, 31, 33, 35, 39, 45, 51, 52, 56, 58, and 59	(263)
2.	Cryotherapy	–	89 (≥18 years, HIV-1positive)	–	HPVtypes 45, 16,18,51, and 58	(269)
		Carbon dioxide as the refrigerant	29	“Double-freeze” procedure of two 3-minute freezes with a thawing interval of 5 min	HPV types 16, 18, 31, 33, 35, 39, 45, 51, 52, 56, 58, 59, 66, 68, 6, 11, 26, 34, 40, 42, 43, 44, 53, 54, 55, 57, 61, 70, and 71	(354)
		–	79 (HIV-positive women)	–	HPV types 16, 18, 31, 33, 35, 39, 45, 51, 52, 56, 58, 59, and 68	(355)
		Liquid nitrogen	3 months old child	–	^*^	(356)
		Carbon dioxide as the refrigerant	34	“Double-freeze” procedure of two 3-minute freezes with a thawing interval of 5 min	Pap smear test irrespective of HPV type
3.	Loop Electrosurgical Excision Procedure (LEEP)	Electric current is passed through a loop of wire, that is used as scalpel to remove the tissue	195	Removal of epithelium and a small amount of underlying stroma from the entire cervical transformation zone	HPV types 16, 18, 31, 33, 35, 39, 45, 51, 52, 56, 58, 59, 66, and 68	(357)
			354		^*^	(358)
4.	Cytotoxic agents	Imiquimod (3.75% or 5% crea	–	Topical application	–	(359)
		Podofilox 0.5% solution or gel			
		Sinecatechins 15% ointment			
		Bichloracetic acid 80%−90%			
		Trichloroacetic acid 80%−90%			
		Vidarabine/ 5-fluorouracil (5%)				(360)
			30		^**^	(361)
		5-Fluorouracil (5%)	31	Topical application	^***^	(362)
		Arsenic trioxide	–	Cell culture	HPVpositive HeLa cancer cell line	(363)
					HeLa cells (HPV18 positive)	(364)
		Cisplatin, carboplatin, and oxaliplatin	–		SiHa (HPV 16+) CaSki (HPV 16+), HeLa (HPV 18+), and UT-DEC-1 (HPV 33+) cell lines	(365)
		Imiquimod (5%)	76	Topical application in cream form	HPV types 6, 11, 42, 44, 16, 18, 31, 33, 35, 45, 51, 52, and 56	(274)
			72	250 mg cream	^****^	(366)
5.	Antiviral drugs	Cidofovir Cidofovir	–	Local intratumor injections	HPV types 16 and 18	(367)
				SiHa, Caski, SCC-147, UM-SCC-47, UD-SCC-2 and UM-SCC-104 cell lines	HPV type 16	(368)
			31	Adjuvant therapy (7.5 mg/ml)	HPV types 6, 11, and 16	(369)
		Ribavirin+pegylated interferon- alfa-2b	1 (HIV negative male)	Ribavirin -oral form (400 mg twice / day)interferon- (120 μg subcutaneously once per week)	–	(289)
			1 (with acquired aplastic anemia)	Ribavirin -oral form (400 mg twice/ day) interferon- (10 million IU subcutaneously once per week)	HPV types 31, 52, and 6	(288)
			1 (Human immunodeficiency virus and hepatitis C virus positive)	–	–	(370)
		Cidofovir	12	Topical application	–	(258)
		Acyclovir	1 (Herpes Simplex Virus and Human Papillomavirus coinfection)	Intravenous (600 mg/three times a day)	–	(371)
		AV2® (a combination of carvone, eugenol, geraniol, nerolidol in equal volumes diluted 50% in olive oil	400	Topical application	HPV types 16, 18, 31, 33, 35, 39, 45, 51, 52, 56, 58, 59, 66, and 68
6.	Gene therapy	Herpes simplex virus type 1 thymidine kinase expression	–	HPV positive CaSki and SiHa cells	HPV type 16	(372)
7.	Herbal therapy	Smoke of burned dried fruit of the pine tree	03	Twice a day	–	(373)
		Paiteling	239	Applied 3 days after the end of menstruation (on days 1–4, 8–11 and 15–18)- a total of 12 such applications	HPV16, 18, 31, 33, 35, 45, 51, 52, 53, 56, 58, 59, 66, 68 and CP8304	(374)
		Carrageenan	–	Crosslinked 3% carrageenan beads	high-titer HPV16 pseudo-viruses into HeLa cells	(291)
			141	Carrageenan-based gel	HPV types 6, 11, 40, 42, 44,54, 16, 18, 26, 31, 33, 34, 35, 39, 45,51, 52, 53, 56, 58, 59, 66, 67, 68, 69, 70, 73, 82, 61,62, 71, 72, 81, 83, 84, and 89	(375)
		Youdujing preparation	35	YDJ external lotion	HPV16 and 18	(294)
		Podophyllotoxin (resin mixture from *Podophyllum peltatum* resin mixture from *Podophyllum peltatum*)	27 Women	20% solution	–	(376)
		*Pinelliapedatisecta*	15 mice	Dried rhizome extract (10 mg/kg/day)	CaSkicell line containing HPV16	(377)
8.	Ranpirnase RNase	Obtained from Leopard frog *Rana pipiens*	42 male volunteers	1 mg/ml ranpirnase formulation for topical application	HPV-11 infected A431 human epidermoid carcinoma cells	(302)
9.	RNAi-based therapies	–	–	–	HPV 18 positive HeLa and C4I	(378)
		9 siRNAs against E6/ E7 genes of HPV-16 /18	–	17~22 sense and antisense hairpin oligonucleotide	HPV-positive CaSki (HPV-16) or HeLa (HPV-18) cell lines	(310)
		HPV16 E7 siRNA	–	Chitosan/HPV16 –E7 siRNA complex	CaSki cells constitutively expressing HPV16 E6 and E7	(311)
			–	Chitosan/HPV16 E7 siRNA nanoparticle complex		(379)
10.	Miscellaneous therapies	Oral cimetidine	4 children	30–40 mg/kg daily divided into 3 doses	–	(380)
		Levamisole	40 patients	5 mg/kg on 3 consecutive days fortnightly for 5 months	–	(381)
		*Propionibacterium acnes* or *Coryneobacterium parvum*	28 volunteers	Intradermal application of 0.1 ml culture in one wart, at 30–40 days intervals for a total of up to five times	–	(382)
		Oral zinc sulfate	20 patients	10 mg/kg	–	(383)
		Bacillus Calmette-Guérin Therapy	50 patients	Local application of BCG mixweekly for 6 consecutive weeks	–	(384)
		Cantharidin (Isolated from blister beetle, *Cantharis vesicatoria*)	12 patients	0.7% solution	–	(385)
		Formic acid application	100 patients	85%	–	(386)
		Bleomycin	Forty patients	1 U/mlat 2-week intervals.	–	(387)

* *Diagnosed by histopathological methods*.

** *HPV typing by RFLP*.

*** *Biopsy confirmed case*.

**** *PCR based positive or negative detection*.

Last but not the least this review is a unique/comprehensive review which highlights the various advances in developing vaccines (both prophylactic and therapeutic) and therapeutic regimens along with recent patents coverage on drugs, vaccines and therapeutics against HPV that will help the scientists and medical practitioners to prevent, treat and facilitate the eradication of the malignancies in relation to HPV in an effective and more promising manner. Moreover, the mind of researchers will be more innovative to combine therapeutic vaccines against HPV with radiation and chemotherapy for designing better control measures along with adopting advanced therapeutic approaches to counter HPV infections and the associated cancerous conditions. The advanced information on HPV vaccines and therapeutics presented in this review compilation along with carrying out more researches in futuristic prospects in the right directions would pave way to design and develop suitable prophylactic and therapeutic vaccines, drugs and treatment modalities delivering clinical outcomes in an improved manner and to combat HPV and its cancerous conditions effectively at global level.

## Conclusion and future prospects

This review highlights the advances in designing prophylactic and therapeutic vaccines as well as treatment regimens against human papilloma virus (HPV) to encourage the development of effective vaccines and therapies against this important disease. For prevention of HPV infection, the use of topical microbicides has been explored widely. For instance, like the use of carrageenan to prevent genital infection due to HPV, several patent applications for formulations are pending which comprise several natural components, such as *Aloe vera*, turmeric, *Citrullus colocynthis*, hard sea salt, myrrh, and garlic to treat HPV. More clinical trials are needed to be conducted to explore the utility of topical microbicides. Studies through mathematical modeling have predicted that vaccination can maintain the antibody levels (especially against HPV-16 and 18) at a much higher level than the level reached due to natural infection for a period of minimum two decades after vaccination. So it can eliminate the requirement of booster dose for a long period. Derivation of the consensus sequence for E6 and E7 genes, followed by codon optimization of these genes and their use as immunogen will pave the way for effective prophylactics. Three commercially available prophylactic vaccines show sufficient efficacy; however, attempts to develop next-generation vaccines that are inexpensive, effective, stable, and that show broad cross-neutralizing immunity are in progress. Recombinant vaccine immunogens based on transgenic plants are an attractive and potentially affordable alternative to vaccines by injection. For example, edible plants can be grown locally and distributed easily without special training or equipment. Approved HPV vaccines based on recombinant VLPs of important high-risk HPV L1 major capsid antigen are effective in controlling HPV infections, but do not have therapeutic applications or protect against cutaneous infections. There are studies to evaluate alternative approaches to deliver current vaccine in a safer and affordable way. For example, microneedle deliveries of lyophilized HPV pseudovirions are thermostable and have been tested in a murine model. Also, acceptance of HPV vaccines has been hindered by many factors associated with place of residence, culture, and economics. Administration of these vaccines has been virtually non-existent in developing countries, mainly because of their extremely high costs and the technical challenges of vaccination, which require multiple doses over a 6-month period (388). Also, refrigeration is needed during shipping and storage, introducing logistical difficulties in areas that lack adequate infrastructure. HPV vaccines cost approximately US $450 (Gardasil) and US $495 (Gardasil 9) in the U.S. for the complete course of three injections. Therefore, second-generation HPV vaccines are needed to reduce the costs of vaccine production and increase immunization schedule feasibility.

A subdominant neutralizing epitope in the HPV L2 protein is an alternative approach to produce recombinant VLP antigens. This well-conserved linear neutralizing HPV epitope, which is located at the amino terminus of the L2 protein, may be exposed while the virus is on the basement membrane during infection. Attempts are being made to formulate more stable VLPs for immunization, and in fact, VLPs which are subjected to a disassembly and reassembly process have been found to be more stable, and the patent has been granted to the technology. Another alternative antigen for use in HPV vaccines is the pentameric subunit or capsomere of the L1 protein that has essential neutralizing epitopes to induce an immune response to protect against HPV. Recombinant capsomeres expressed in *E. coli* represent one important approach to reducing manufacturing costs. Studies in animal models have reported that HPV capsomeres alone induce lower antibody titers than VLPs. Therefore, this approach will require further optimization for increased antibody titers. However, the pentamers can be lyophilized for greater thermostability, suggesting the potential for formulations that can be shipped and stored without refrigeration (389). Thus, use of capsomeres is an attractive and affordable option for developing second-generation HPV vaccines (389).

On the other hand, production of combined preventive-therapeutic antigens, focusing on fusions of L2 with E7, or both E6 and E7, is in the early stages, and their therapeutic effectiveness has not been demonstrated. However, these therapeutic vaccines have shown some promise in some early clinical trials. Curcumin has also shown the potential to prevent HPV-associated oral cancers by selectively inhibiting E6 oncogene-mediated p53 degradation. Apart from vaccination, several therapeutic approaches are also becoming popular nowadays to counter HPV, and if given in conjugation with vaccines, the disease may be combated in a better way. Among some treatment strategies, a photodynamic therapy which uses 5-aminolevulinic acid has been tested against HPV-6, 11, 16, and 18 and a higher clearance rate (83.9%) has been observed, without affecting the cervical tissue morphology. Topical application of few cytotoxic compounds including podophyllin, arsenic trioxide, carboplatin or trichloroacetic acid also has been suggested to remove genital warts. Interferons and immunomodulators like imiquimod also have been tested for their efficacy in removing genital warts and found useful. Antivirals like cidofovir, ribavirin, and acyclovir have been tested against HPV, but cidofovir has been used widely, and since it can be administered through inhalation, additional benefits are present for using it in clinical applications. Ranpirnase RNase enzyme cleaves dsRNAs, and in the form of a topically applicable ointment, it has proved effective in treating anal warts. The treatment modules encompassing the use of antisense RNA are still in infancy but have shown some promise in therapeutic efficacy.

For HPV vaccines, pre-adolescent girls are the primary target but evaluation of the cost effectiveness of vaccination of other groups needs to be done. For certain candidate vaccines entering phase III trial, a viable vaccine (prophylactic) against a few types of HPV may become available in a less period of five years. For complementing conventional therapy designing of the therapeutic vaccines (under investigation) are done mostly. However, the extent of benefit or the cost at which such benefits can be offered to the women are not yet clear. Clearing of the earliest stage of infection due to HPV by therapeutic vaccines is possible but is less developed. Certainly such vaccines hold promises to reduce the suffering as well as cost of treatment associated with disease of the cervix. For precancerous lesions, it is, however, crucial to continue the process of development of accurate screening as well as treatment plans and programs as there is forward movement of the process of vaccine development.

No one can deny the fact that vaccination against HPV has proven to be a landmark in the history of prevention of cancer. The incidence of HPV-induced cervical cancer will reduce drastically if adolescent girls are immunized, and this must be the priority not only in the industrialized world, but also in developing nations where it is sometimes impossible to detect the precancerous lesions at an early stage. It is however unfortunate that even in the developed nations of the Western world, the screening to detect cervical HPV-induced cancer is not performed on a regular basis. In this context, implementation of immunization of girls in every nation universally is of immense benefit. To succeed in such aspect of prevention of HPV infection especially in women, convincing the parents as well as their daughters is a top priority. Similarly, vaccination in homosexual males is of utmost relevance concerning prevention of HPV in the community. But to assess the sexual orientation in every male is somewhat difficult for which universal male vaccination is essential. With the usage of efficient vaccination and application of potent therapeutics, the effective elimination of the disease is expected and in fact combinations of several therapies have helped people in getting rid of this ailment. For all these reasons, it is necessary for the public health authorities to remain proactive and at the same time innovative methods will also be required for financing introduction of HPV vaccine and advancing the development of potent therapeutics.

## Author contributions

All the authors substantially contributed to the conception, design, analysis, and interpretation of data, checking and approving the final version of the manuscript, and agree to be accountable for its contents. MD, KD, SH, and RT initiated this review compilation. SC, RK, AM, MP, and DK updated different vaccines and therapies. RK designed table. KK designed the figures. KD, HI, MD and WC reviewed, analyzed and edited.

### Conflict of interest statement

The authors declare that the research was conducted in the absence of any commercial or financial relationships that could be construed as a potential conflict of interest.
